# Inter-trial effects in priming of pop-out: Comparison of computational updating models

**DOI:** 10.1371/journal.pcbi.1009332

**Published:** 2021-09-03

**Authors:** Fredrik Allenmark, Ahu Gokce, Thomas Geyer, Artyom Zinchenko, Hermann J. Müller, Zhuanghua Shi

**Affiliations:** 1 Department of Psychology, Ludwig-Maximilians-Universität München, Munich, Germany; 2 Department of Psychology, Kadir Has University, Istanbul, Turkey; University of Tokyo: Tokyo Daigaku, JAPAN

## Abstract

In visual search tasks, repeating features or the position of the target results in faster response times. Such inter-trial ‘priming’ effects occur not just for repetitions from the immediately preceding trial but also from trials further back. A paradigm known to produce particularly long-lasting inter-trial effects–of the target-defining feature, target position, and response (feature)–is the ‘priming of pop-out’ (PoP) paradigm, which typically uses sparse search displays and random swapping across trials of target- and distractor-defining features. However, the mechanisms underlying these inter-trial effects are still not well understood. To address this, we applied a modeling framework combining an evidence accumulation (EA) model with different computational updating rules of the model parameters (i.e., the drift rate and starting point of EA) for different aspects of stimulus history, to data from a (previously published) PoP study that had revealed significant inter-trial effects from several trials back for repetitions of the target color, the target position, and (response-critical) target feature. By performing a systematic model comparison, we aimed to determine which EA model parameter and which updating rule for that parameter best accounts for each inter-trial effect and the associated *n*-back temporal profile. We found that, in general, our modeling framework could accurately predict the *n*-back temporal profiles. Further, target color- and position-based inter-trial effects were best understood as arising from redistribution of a limited-capacity weight resource which determines the EA rate. In contrast, response-based inter-trial effects were best explained by a bias of the starting point towards the response associated with a previous target; this bias appeared largely tied to the position of the target. These findings elucidate how our cognitive system continually tracks, and updates an internal predictive model of, a number of separable stimulus and response parameters in order to optimize task performance.

## Author summary

In many perceptual tasks, performance is faster and more accurate when critical stimulus attributes are repeated from trial to trial compared to when they change. Priming of pop-out (PoP), visual search with sparse search displays and random swapping of the target feature between trials, is a paradigm in which such inter-trial effects can be traced back over several recent trial episodes. While many studies have explored PoP paradigms, the mechanisms underlying priming of the search-critical target feature, the target position, and the response-critical information are not yet fully understood. Here, we addressed this question by applying evidence accumulation (EA) decision models to the data from a previously published PoP study. The modeling framework combines evidence accumulation with Bayesian updating of the model parameters. Comparison of (> 1000) different combinations of decision models and updating rules revealed that the featural and positional priming effects were best explained by assuming that attentional “weight” resources are dynamically redistributed based on the recent history of target color and position, whereas response decisions are biased based on the recent history of the response-critical property of targets occuring at a particular (and nearby) position(s). These findings confirm that our cognitive system continually tracks, and updates an internal predictive model of, a number of separable stimulus and response parameters in order to optimize task performance.

## Introduction

Selecting the most relevant, and deprioritizing irrelevant, visual information is important in many everyday tasks. These priorities need to be flexibly updated based on current goals as well as on prediction about what visual dimensions and features will need to be attended to and processed in pursuing these goals. Visual search tasks provide a rich source of evidence for studying the dynamics underlying this updating: Apart from more general goal-based, top-down adjustments of the task set, the system settings are constantly adapted in response to the stimuli encountered on sequential search trials. These adaptations give rise to inter-trial, or selection, history effects, where performance on the task improves when some critical stimulus property is repeated across trials and impaired if it changes (e.g. [1]). Such inter-trial effects have been documented consistently for cross-trial repetitions/switches of specific target-defining features, such as color, spatial frequency [2,3], and size [4], for whole target-defining dimensions (e.g. [5,6]), and for target position [7,8]. Importantly, models of the updating of attentional control settings make predictions about these inter-trial effects that can be examined against the data (e.g. [9,10]), thus helping us understand the underlying computational principles. Accordingly, the goal of the present study was to apply mathematical modeling–specifically, evidence accumulation models to RT data to a ‘classical’ visual singleton, or ‘pop-out’, search task (adapted from [3,8]), employing a novel approach, namely: taking into account observers’ natural perceptual uncertainty in performing a given task and, associated with this, the possibility of adaptive trial-by-trial updating of the model parameters, in order to characterize the perceptual and cognitive mechanism/s of multiple types of (non-/spatial) inter-trial ‘priming’ in this task.

Paradigms examining for inter-trial effects differ with regard to how long-lasting the respective (feature-, dimension-, position-specific) inter-trial effects are. One paradigm that has been documented to produce inter-trial effects of longer durations is known as ‘*priming of pop-out*’ (PoP; e.g. [2,3,8,11]). In a previous modeling study [12], we used a paradigm in which a pop-out target was presented in a dense search array (consisting of 39 closely spaced items) and the features of the (homogeneous) distractor items never changed across trials. In that study, described in more detail below, we only found significant inter-trial effects from a single trial back. By comparison, the PoP paradigm produces inter-trial effects that can be traced back for approximately five to eight trials for (repetitions/switches of) the target-defining feature [3] as well as for the target position [8,13]. PoP studies typically use sparse search displays (e.g., with three, widely spaced items only) and, importantly, random swapping across trials of the search-critical target and distractor features (e.g., the target may be red amongst green distractors on one trial, and green amongst red distractors on the next trial). Of note, feature-based inter-trial effects manifest specifically with sparse displays and target-distractor color swapping, but not or to a lesser extent with dense displays or constant distractor color [13,14]. That is, in PoP paradigms, guidance based on the computation of local feature-contrast, the major determinant of bottom-up saliency (e.g., see [15]), is relatively ineffective (because there is little *local* feature contrast), and in contrast to the name ‘priming of *pop-out*’, the target actually fails to be the first item to draw attention in a significant proportion of trials (e.g., Rangelov et al. [16] estimate this proportion to be of the order of 20% to 70%, consistent with eye-movement evidence such as from Becker [17]). Given that (bottom-up) saliency coding is a relatively non-reliable guide to finding the target, and that the target is consistently defined in the color dimension, the search-guidance system comes to rely on other types of information to optimize target selection under PoP conditions: in particular, top-down–color-feature-based–guidance processes, along with reliance on positional information (also evidenced by eye movements often returning, on trial n, to the target location on trial n-1). The roles of these, while traceable in dimension-based paradigms (feature-based especially with color-defined targets; e.g., [5]), become much more prominent under PoP conditions, and empirically, they exhibit a more persistent (n-back) effect than dimension-based priming effects. Thus, the underlying mechanisms, that is, the neural machinery that is primed, are likely to be different. Accordingly, attempting to model these (feature- and position-based inter-trial) effects using our modeling framework provides a theoretically interesting extension to our previous study.

Further, feature-based inter-trial effects in PoP paradigms are typically independent of, or additive to, positional intertrial effects [8,18]. Some PoP studies also found inter-trial effects for the response-critical target feature, such as whether a section (“notch”) of the (color pop-out) target is cut off at the top or the bottom [19–21], while other studies failed to find such effects [3,22]. Interestingly, Gokce et al. [19] found the effect of response repetition to be dependent on position repetition: response repetition, from one trial back, expedited RTs significantly only when the target position was also repeated (similar results have also been found by [23–25]). One reason why some studies failed to find significant inter-trial effects for the response feature may be that target position repetitions were relatively rare. For instance, in the studies by Maljkovic et al. [3,22], the target occurred at one of twelve positions, randomly chosen from trial to trial; that is, the target position would have repeated on only some 8% of the trials, and there would have been a response repetition on only half these trials, making it hard to resolve the interaction.

**Fig 1:**
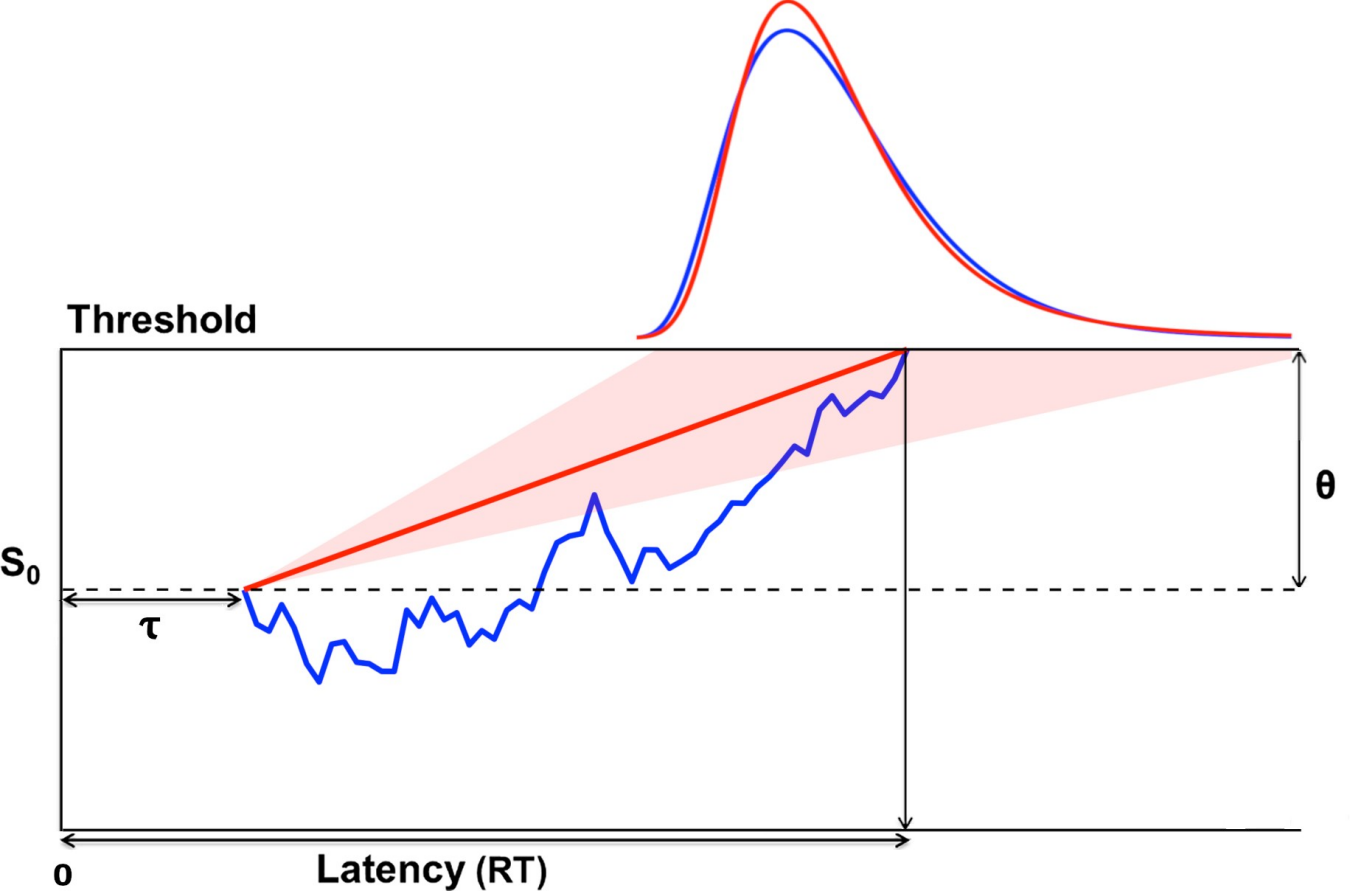
Evidence accumulation models. The DDM assumes that evidence accumulates, from the starting point (S_0_), through random diffusion in combination with drift at a constant rate r until a boundary (i.e., threshold, θ) is reached (illustrated in blue). The Linear Approach to Threshold with Ergodic Rate (LATER) model (illustrated in red) makes the same assumptions, except that there is no random diffusion, but instead the rate r varies across trials (so as to explain trial-to-trial variability in RTs). In addition, a non-decision time (NDT) *τ* is added to the boundary crossing time on each trial, to capture time spent on everything else than the perceptual decision (e.g., the time to prepare and execute a selected motor response).

While there have been many previous studies on inter-trial effects in visual search, most of these have focused on mean response times (RTs) (and error rates). However, more information can be obtained from the shape of the RT distribution. For a variety of different tasks, the shapes of RT distribution shapes are well predicted by evidence accumulation models [26,27], with the ‘drift-diffusion model’ (DDM) being one particularly influential model of this type [28–30]. The DDM assumes that a decision between two different perceptual hypotheses is made by accumulating multiple pieces of evidence over time, summing the logarithm of the likelihood ratio, under the two different hypothesis, for these pieces of evidence, and making a decision when the sum reaches a threshold (in either direction; see Fig 1). This model has four important parameters: the drift rate, that is, a tendency to, on average, drift towards one or the other boundary (representing the average strength of the pieces of noisy evidence in favor of the respective hypothesis); the separation between the decision boundaries (boundary separation); a starting point (representing any initial bias, e.g., from a-priori priors or selection history); and a non-decision time (NDT, representing time spent on processes that are not part of the perceptual decision making as such, such as preparation and execution of the motor response). Another evidence accumulation model is the ‘Linear Approach to Threshold with Ergodic Rate’ (LATER) model [31,32]. This model also assumes that evidence accumulates until it reaches a threshold, but, unlike the DDM, it assumes that during a single perceptual decision, evidence accumulates at a constant rate, with this rate varying randomly across search episodes (see Fig 1).

In a previous study [12], we fit the DDM and LATER models to RT distributions from three different pop-out visual search experiments, in order to examine which model parameters were involved in different types of inter-trial effects occurring in the respective paradigms. In two of these experiments, we used a block-wise frequency manipulation of the response-critical feature (RCF) of the search displays. In one of these experiments, the response was made based on whether a target was present or absent, and in the other based on whether the target was defined in the color or the orientation dimension. In order to model inter-trial effects, we assumed that the starting point and the drift rate of the evidence accumulation model each could change after each trial based on two stimulus properties on that trial: the RCF and the target-defining dimension (TDD). We considered a number of different updating rules, different plausible rules of how these parameters could change, such that the RCF and TDD each could influence either the starting point or the drift rate (or neither) of the evidence accumulation process, and compared these in a factorial model comparison. This comparison revealed that the best combination of updating rules for the RCF and the TDD (in terms of the Akaike Information Criterion, AIC) involved updating the starting point based on the RCF, using a form of Bayesian updating consistent with an interpretation of the starting point as the logarithm of the prior odds, in this case the prior odds of the target being present versus absent or of being defined by color versus orientation; and updating the drift rate based on the TDD, using a ‘weighted-rate’ updating rule consistent with the ‘dimension-weighting account’ [5,6]. This model captured both the effects of the probability manipulation and the inter-trial effects based on the RCF and the TDD quite well. However, the inter-trial effects were of relatively short duration: significant inter-trial effects were resolvable only from a single trial back, as is generally the case for ‘dimension-weighting’ studies [14]. Accordingly, we could not draw any strong conclusions about how well our model would capture the dynamics of longer-lasting inter-trial effects described in the literature [33,34] and the decay of the memory traces underlying these inter-trial effects over time. In the present study, we aimed to address this question by applying our modeling framework to the PoP paradigm which is known to produce longer lasting inter-trial effects.

As elaborated above, each type of inter-trial effect–faster RTs with repetition, compared to change, of the target-defining feature, the target position, and the response-critical feature–could be mediated by different mechanisms, such as more efficient target processing or responding based on less evidence (i.e., a response bias). Depending on the underlying mechanism, one can make different predictions about how RT distributions will differ across inter-trial conditions, such as repetition versus switch of the target-defining color. In our modeling framework, such differences in RT distributions across different inter-trial conditions are predicted by changes in the evidence accumulation model parameters based on stimulus and selection history. Thus, by comparing different updating rules, which differ in terms of which model parameters change based on the history of each stimulus attribute, and finding the updating rule that best explains the RT distributions, we can make inferences about the likely underlying mechanism for each type of inter-trial effect. To this end, we applied this approach to the data set collected by Gokce et al. [19], which revealed all three types of inter-trial effects considered above: faster RTs for repetitions (vs. changes) of the target-defining feature (color: green vs. red), the target position, and the response-critical feature (the position of a “notch”, top vs. bottom, in the target item).

**Fig 2:**
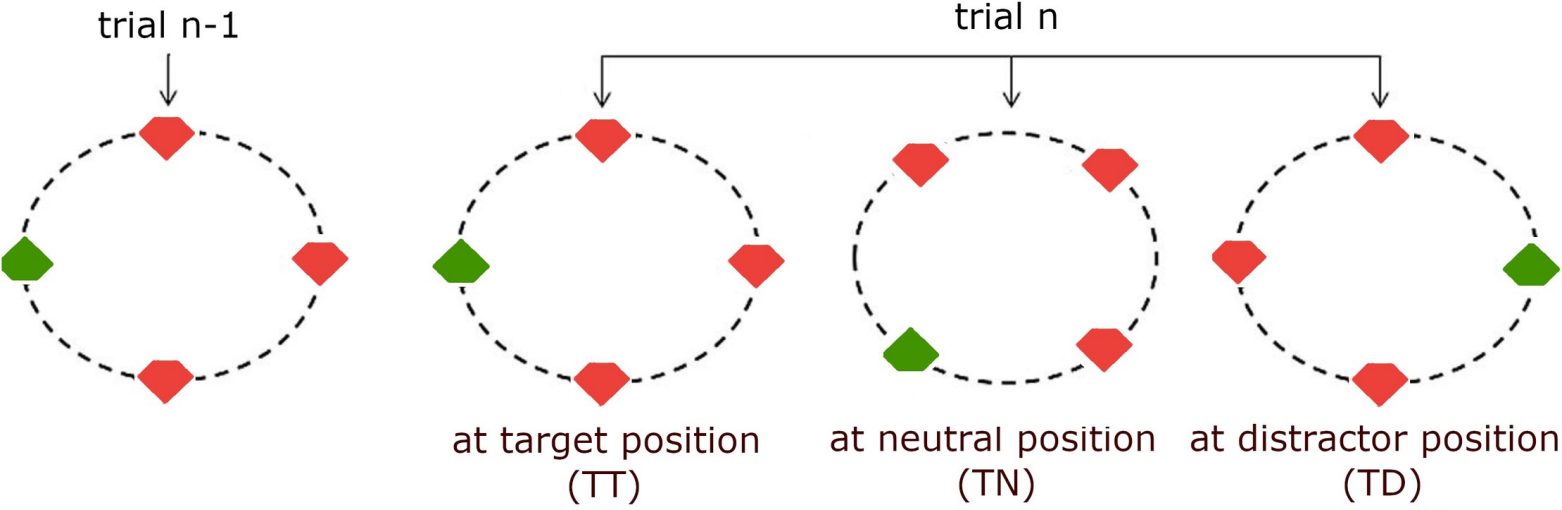
Illustration of the stimulus. Illustration of the search display and the positional inter-trial transitions. The circles were not part of the actual stimulus, but are only shown to illustrate how the items were positioned on a circle. On each trial, four out of eight possible locations were occupied by the search items, allowing the items to form either a square or a diamond arrangement.

Fig 2 illustrates the stimuli and inter-trial transitions used in Gokce et al. [19]. In that study, participants were required to find a singleton target stimulus defined by an odd-one-out color (the only green item amongst red items, or the only red item amongst green items) and respond according to whether the target had a cut-off section (notch) at the top or the bottom. The target and distractor stimuli were positioned on a virtual circle, forming either a regular square or diamond arrangement on a given trial. Across consecutive trials *n-1* and *n*, the target/distractor color polarity could either repeat (e.g.: trial *n-1*: red target amongst green distractors → trial *n*: red target amongst green distractors) or switch (e.g., trial *n-1*: red target amongst green distractors → trial *n*: green target amongst red distractors), with equal probability. Independently of this, the target on a given trial (*n*) could appear at the same location as on the previous trial (*n-1*), henceforth referred to as a TT trial; it could appear at a previously empty, or neutral location, henceforth labelled TN trial; or it could occur at a previous distractor location, TD trial; again, all three positional inter-trial transitions (see Fig 2 for an illustration) were equally likely. Finally, the ‘orientation’ of the response-critical notch in the target item (top or bottom) could be either repeated or switched across consecutive trials, again with equal probability. Given this, the data allowed for the examination of target feature-, position-, and response-based inter-trial priming effects; note that with regard to positional intertrial priming, the design made it possible to dissociate facilitatory (comparison TT vs. TN) and inhibitory (TN vs. TD) priming with respect to the neutral (TN) condition.

In summary, by comparing different models of how RTs are affected by stimulus history, we aimed to address a number of questions. The primary aim was to examine which types of updating can better predict the temporal profile of *n*-back inter-trial effects depending on whether some target feature (i.e., the target color and/or the ‘orientation’ of the notch), or the position, was repeated or changed. In addition, we aimed to investigate the degree of spatial specificity of the inter-trial effects for the response-critical target feature and the target-defining feature (here color), by comparing updating rules with differing degrees of position specificity of the parameter updates. Finally, by comparing updating rules operating on different parameters of the evidence accumulation model, we also aimed to contribute evidence towards understanding the underlying mechanism for each type of inter-trial effect. Different updating rules implement different hypotheses about what type of memory is carried over across trials and causing the priming effects. This may be a stimulus-response (S-R) binding carried over from a previous trial, resulting in a bias towards repeating the same response or perceptual decision (e.g., about the target location), or it may be a shift of attentional weight towards the target location or feature that is carried over across trials. Thus, by finding the best updating rule for each type of inter-trial effect, we aimed to clarify both the nature of the memory responsible for the inter-trial effect, the spatial specificity of this memory, and the speed of memory decay.

## Results

### Behavioral results

**Fig 3:**
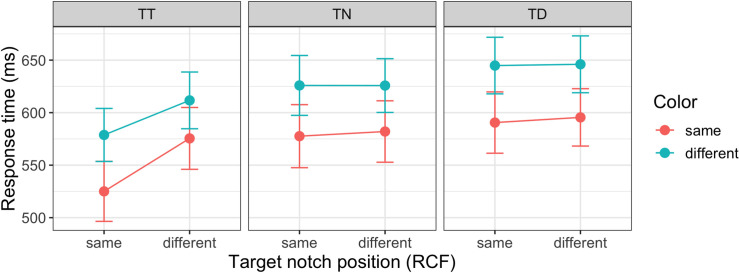
Response times. Mean response times for repeated/non-repeated color, target notch ‘orientation’ (the response- critical feature), and for the different positional transitions: target at previous target position (TT), target at previously neutral (i.e., empty) position (TN), and target at previous distractor position (TD). Error bars show the 95% confidence intervals. RCF: response-critical feature.

Fig 3 depicts the mean RTs as a function of the response-critical target notch position (i.e., effectively, the response: same vs. different relative to the preceding trial) for trials with a repetition versus a switch of the target-defining color (same vs. different) across consecutive trials, separately for the three inter-trial target location transitions (target at previous target location, TT vs. at previously empty neutral location, TN vs. at previous distractor location, TD). A repeated-measures ANOVA with color (repetition/switch), response (repetition/switch), and target position (TT, TN, TD) as factors revealed all main effects to be significant (response: F(1, 13) = 7.75, p = .015, $\eta_{p}^{2}$ = 0.37, BF_incl_ > 1000; color: F(1, 13) = 160.9, p < .001, $\eta_{p}^{2}$ = 0.93, BF_incl_ > 1000; position; F(1.2, 15.6) = 72.82, p < .001, $\eta_{p}^{2}$ = 0.85, BF_incl_ > 1000, Huynh-Feldt corrected degrees of freedom). RTs were faster when the target-defining color repeated vs. changed (48-ms difference), and when the response-critical notch position repeated vs. changed (16-ms difference). And RTs were significantly faster when the target appeared at the same position compared to either a previous distractor location (TT vs. TD: 46-ms difference, Bonferroni-corrected t(13) = 9.28, p_bonf_ < .001, BF_10_ > 1000) or a previously empty (neutral) location (TT vs. TN: 30-ms difference, t(13) = 7.15, p_bonf_ < .001, BF_10_ > 1000); there was also a significant cost when the target appeared at a previous distractor position vs. a previously empty (neutral) position (TD vs. TN: –16-ms difference, t(13) = –9.48, p_bonf_ < .001, BF_10_ > 1000). In addition, the interactions RCF × position (F(1.4, 19) = 23.3, p < .001, $\eta_{p}^{2}$ = 0.64, BF_incl_ > 1000 and RCF × color (F(1,13) = 6.19, p = .027, $\eta_{p}^{2}$ = 0.32, BF_incl_ = 0.56) were significant, although the RCF × color interaction was not supported by the Bayesian analysis. Repeated-measures ANOVAs conducted separately for each positional inter-trial transition condition with response (repetition/switch) and color (repetition/switch) as factors revealed that the effects involving response (position of the target notch) were significant only in the repeated target position (TT) condition (main effect of response: F(1,13) = 32.6, p < .001, $\eta_{p}^{2}$ = 0.72, BF_incl_ > 1000, response × color interaction: F(1,13) = 6.0, p = .029, $\eta_{p}^{2}$ = 0.32, BF_incl_ = 0.89), but not in the TN or TD conditions (all *p* > .3).

### Model comparison results

**Fig 4:**
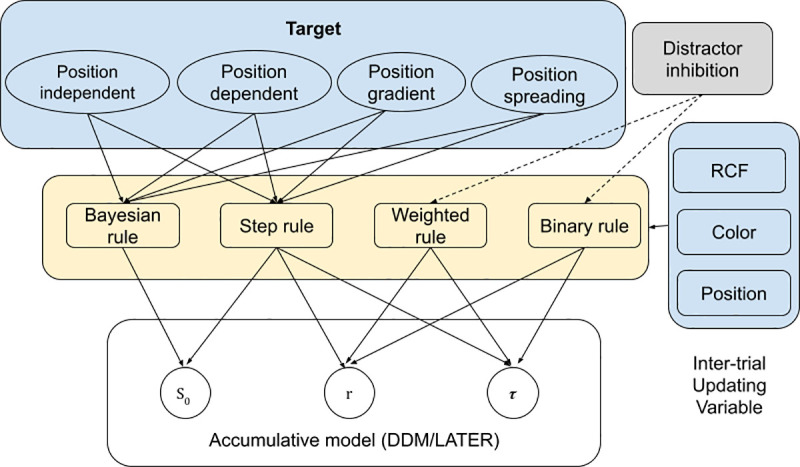
Illustration of the hierarchical modeling framework. Each hierarchical model consists of an evidence accumulation model (either the drift-diffusion model or the LATER model) and either no updating or one updating rule for each parameter of the evidence accumulation model (starting point S_0_, evidence accumulation rate r, and non-decision time *τ*). Each updating rule belongs to one of the four categories shown in the middle layer of the figure, and is applied to one of the three inter-trial updating variables shown in the blue box on the right side of the figure: the response-critical feature (RCF), the target-defining color, or the target position. For some of the updating rules based on the RCF or color, there are three different versions of the rule, differing in their degree of position-specificity (top level of the hierarchy). These rules could be fully position-independent (PI), fully position-dependent (PD), with a gradient-like dependence on the change of position (PG), or starting out fully position-dependent but then spreading (PS). See the main text for detailed descriptions.

In our modeling framework (see Fig 4), we treat each trial of the experiment as a perceptual decision, which is modeled as an evidence accumulation process, and we allow the parameters of that evidence accumulation process (i.e., the starting point, rate of evidence accumulation, and non-decision time) to change from trial to trial based on recent stimulus history.

In particular, we consider three aspects of stimulus history: the cross-trial history of the response-critical feature (RCF), of the target color, and of the target position. For each of these, we consider different updating rules, each implementing a possible way in which one model parameter could change based on this aspect of stimulus history (see detailed mathematical description in the section of “Models and updating rules”). The aspect of stimulus history that an updating rule is based on is referred to as the updating variable (UV) of that rule. Each of the updating rules belongs to one of four categories: Bayesian rules, step rules, weighted rules, and binary rules. Binary rules have two different values of the updated parameter, one on trials where the UV was repeated from the immediately preceding trial (*n-1*) and the other on trials where it changed. Binary rules were included for comparison, serving as a ‘baseline’ to assess how much better inter-trial effects could be explained when taking into account trial history further back than *n-1*; step rules, weighted rules, and Bayesian rules represent three different ways of doing this. Step rules assume that repetition effects were partially carried over to future trials. For example, if the evidence accumulation rate was faster because the target color had been repeated between trial *n-1* and trial *n*, some of this repetition benefit would be carried over to trial *n+1* (and a smaller proportion carried over to trial *n+2* and so on). Weighted rules instead assume that each state of the UV had an associated weight which determined the value of the updated parameter on trials where that state of the UV occurred. After each trial, some of the weight was shifted to the state of the UV which occurred on that trial, in such a way that the total weight remained constant, as if a limited resource was being reallocated. There was also memory decay of previous weight reallocations. The Bayesian rules were applied specifically to the starting point parameter, and assumed that the relative frequencies of the different states of the UV were learned through Bayesian updating, with memory decay as in the dynamic belief model of Yu and Cohen [35]. These frequencies were assumed to define a prior for the evidence accumulation process on each trial, implemented by setting the starting point to the logarithm of the prior odds of the state of the UV on that trial. For some of the updating rules based on the RCF and the target color, we also compared four different versions of the rule, differing in their degree of position specificity as well as in whether memories learned at a particular position would spread over time. These rules could be fully position independent (PI), fully position-dependent (PD), with a gradient-like dependence on the change of position (PG), or initially fully position-dependent but with spreading over time (PS). The PI rule assumes that the influence from RCF or color on previous trials does not depend at all on whether the target position was the same or had changed, while the PD rule assumes no influence at all from a previous trial unless the same target position is repeated. By contrast, the rule of PG suggests a stronger influence from a previous trial the closer the target is to its previous position. Finally, the PS rule assumes that learning is initially fully position specific but later spreads (e.g. because the exact position in which a particular target appeared is gradually forgotten). For some of the position-based updating rules, there are three different versions, with and without inhibition of previous distractor locations which could be either fully matched with target location facilitation so that weight is only transferred from distractor to target locations, or involve separate processes of transferring weight to the target and away from distractors (including to and from empty locations). Further details about the updating rules and the model fitting are provided in the Methods and models section below.

We compared different updating rules based on how well the model, when using this updating rule, predicts RTs on all trials and determined the best of these updating rules in terms of the Akaike Information Criterion (AIC; see Methods and models section for more details). The AIC is a measure of the quality of a model, which takes into account goodness of fit (as measured by the likelihood) and also penalizes models with more free parameters. Lower AIC values indicate better model performance. In total, we compared 12 different updating rules for updating based on the response-critical feature, eight for the target color and ten for the target position (taking into account also the two different evidence accumulation models DDM and LATER, there were thus a total of 2*12*8*10 = 1920 possible models).

**Fig 5:**
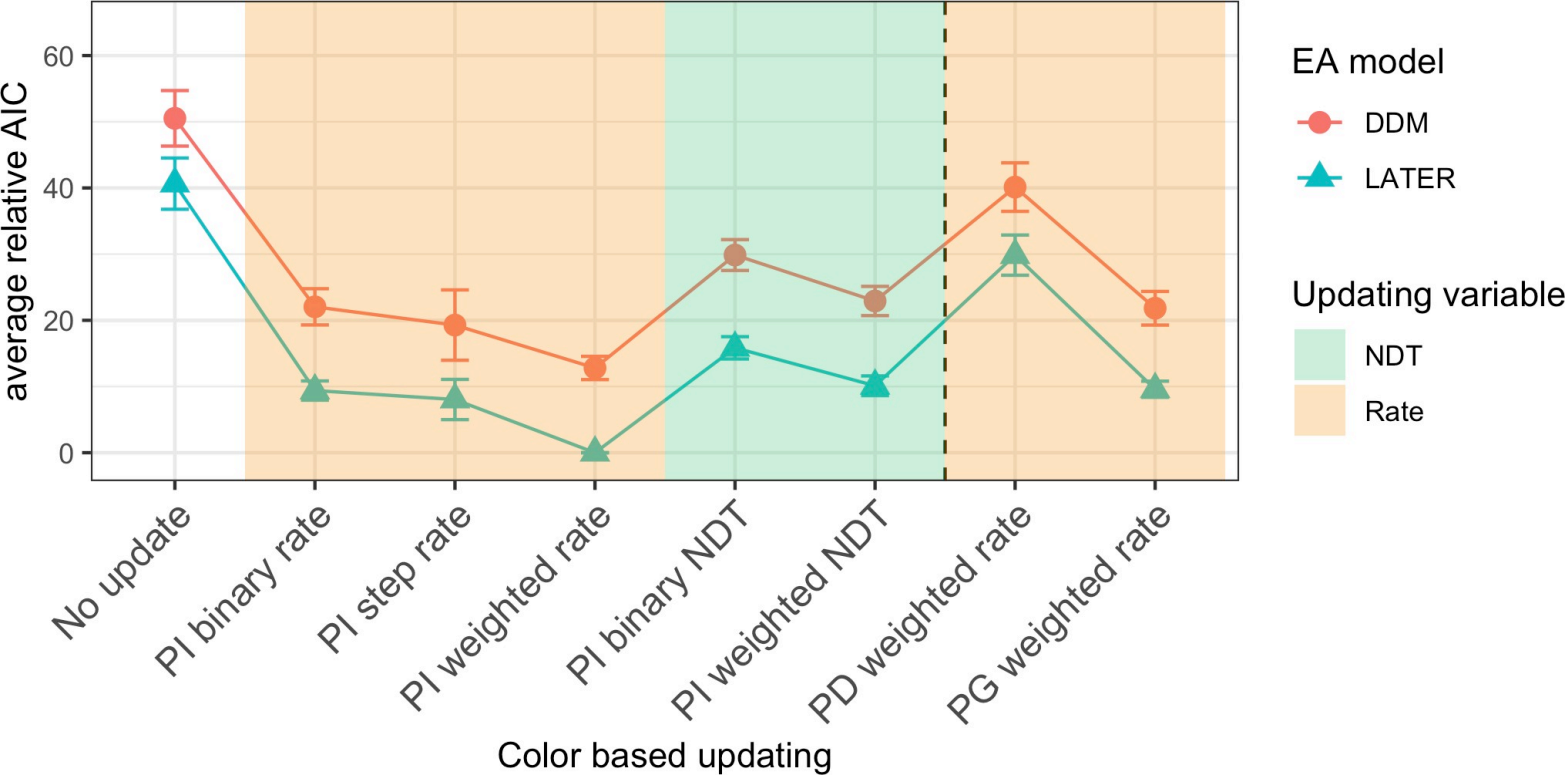
Model comparison for color-based updating. Mean relative AIC for all different color-based updating rules when using the best updating rules for response-critical feature (RCF) and position. The dashed vertical line separates rules that update based on color alone (left) and rules that also take the target position into account (right). The different background colors mark rules that update either the rate (orange) or the non-decision time (green). Error bars represent the standard error of the mean over participants and sessions. PI: position-independent; PD: full position-dependent; PG: a gradient-like dependence on the change of position; NDT: Non-decision time.

**Fig 6:**
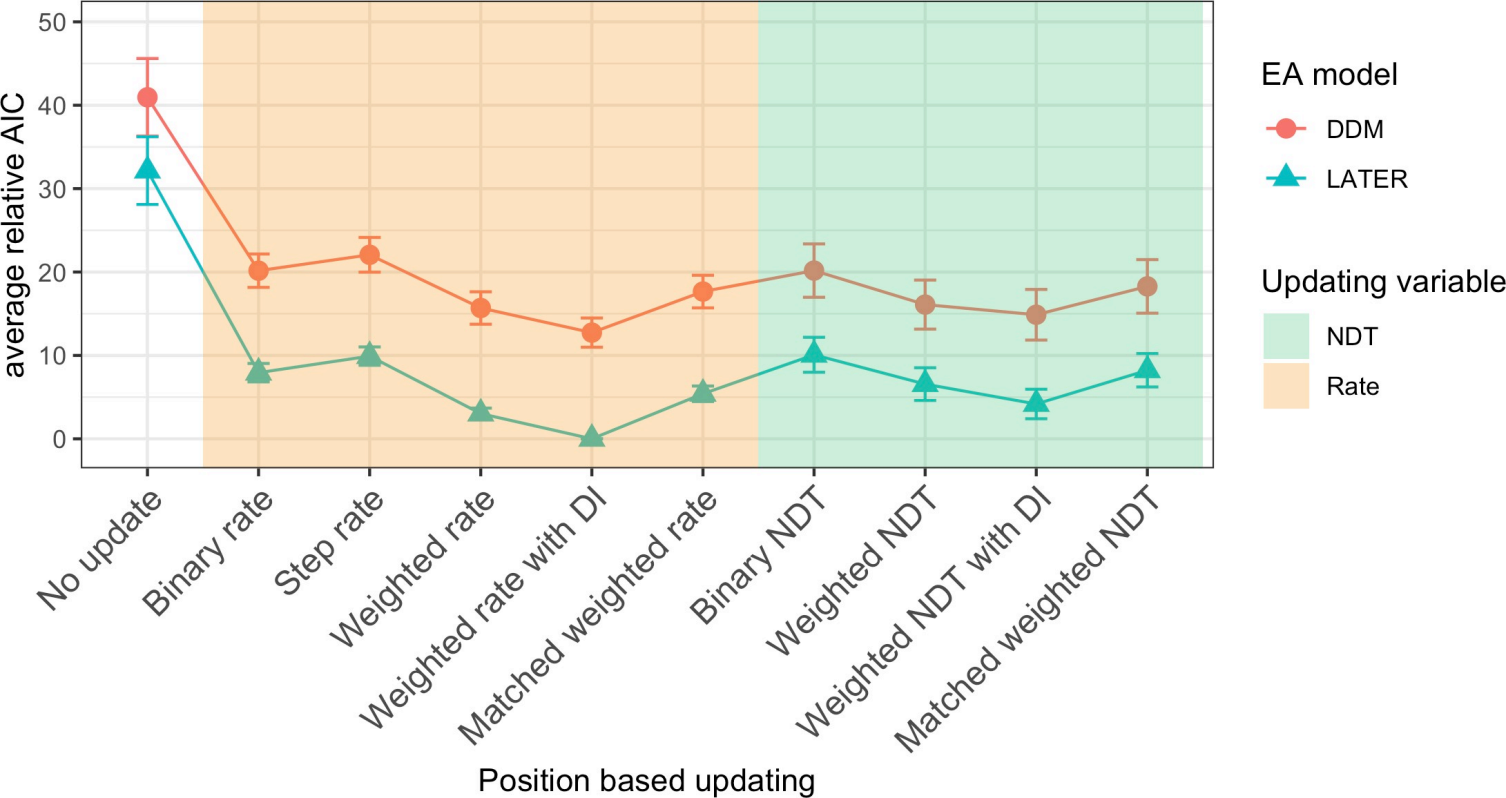
Model comparison for position-based updating. Mean relative AIC for all different position-based updating rules when using the best updating rules for response-critical feature (RCF) and color. The different background colors mark rules that update either the rate (orange) or the non-decision time (green). Error bars represent the standard error of the mean over participants and sessions. DI: distractor inhibition; NDT: Non-decision time.

**Fig 7:**
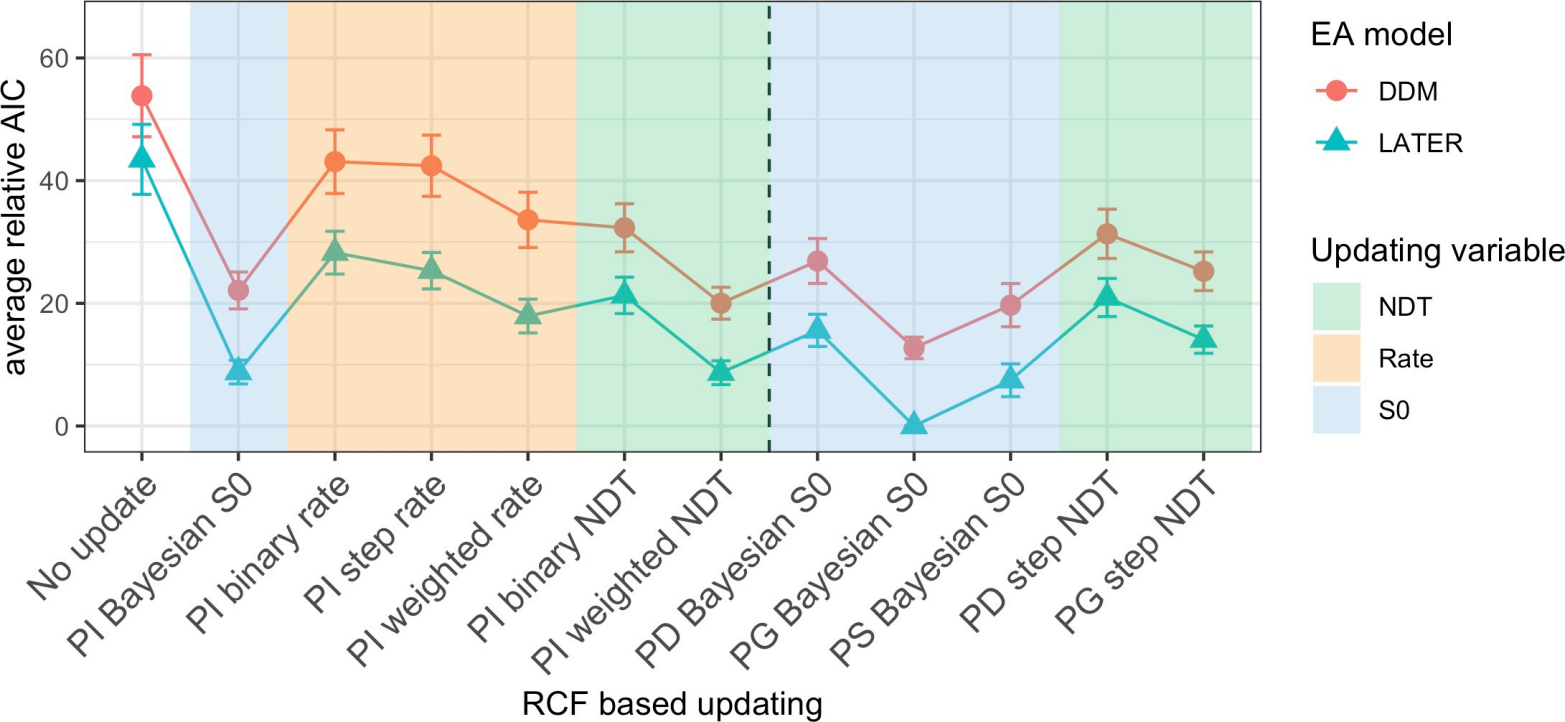
Model comparison for response feature-based updating. Mean relative AIC for all different RCF-based updating rules when using the best updating rules for color and position. The dashed vertical line separates rules that update based on the RCF alone (left) and rules that also take the target position into account (right). The different background colors mark rules that update either the starting point (blue), the rate (orange) or the non-decision time (green). Error bars represent the standard error of the mean over participants and sessions. PI: position-independent; PD: fully position-dependent; PG: a gradient-like dependence on the change of position; NDT: Non-decision time; S0: Starting point.

Figs 5, 6 and 7 show the mean relative Akaike Information Criteria (AIC) for each of the response feature-, target color-, and target position-related updating rules. For each of these three updating variables, the AIC for each updating rule was evaluated for a model which used the best updating rule, in terms of having the lowest associated AIC, for each of the other two updating variables (see Methods and models section for further details). For each individual participant and session, we subtracted the AIC of the overall winning model (based on all participants and sessions) from the AIC of every other model for that participant and session, and finally we averaged this relative AIC across all participants and sessions. This resulted in a relative AIC of zero for the winning model, while the relative AIC of other models indicate how much worse they are compared to the winning model.

#### Target color-based updating models

**Fig 8:**
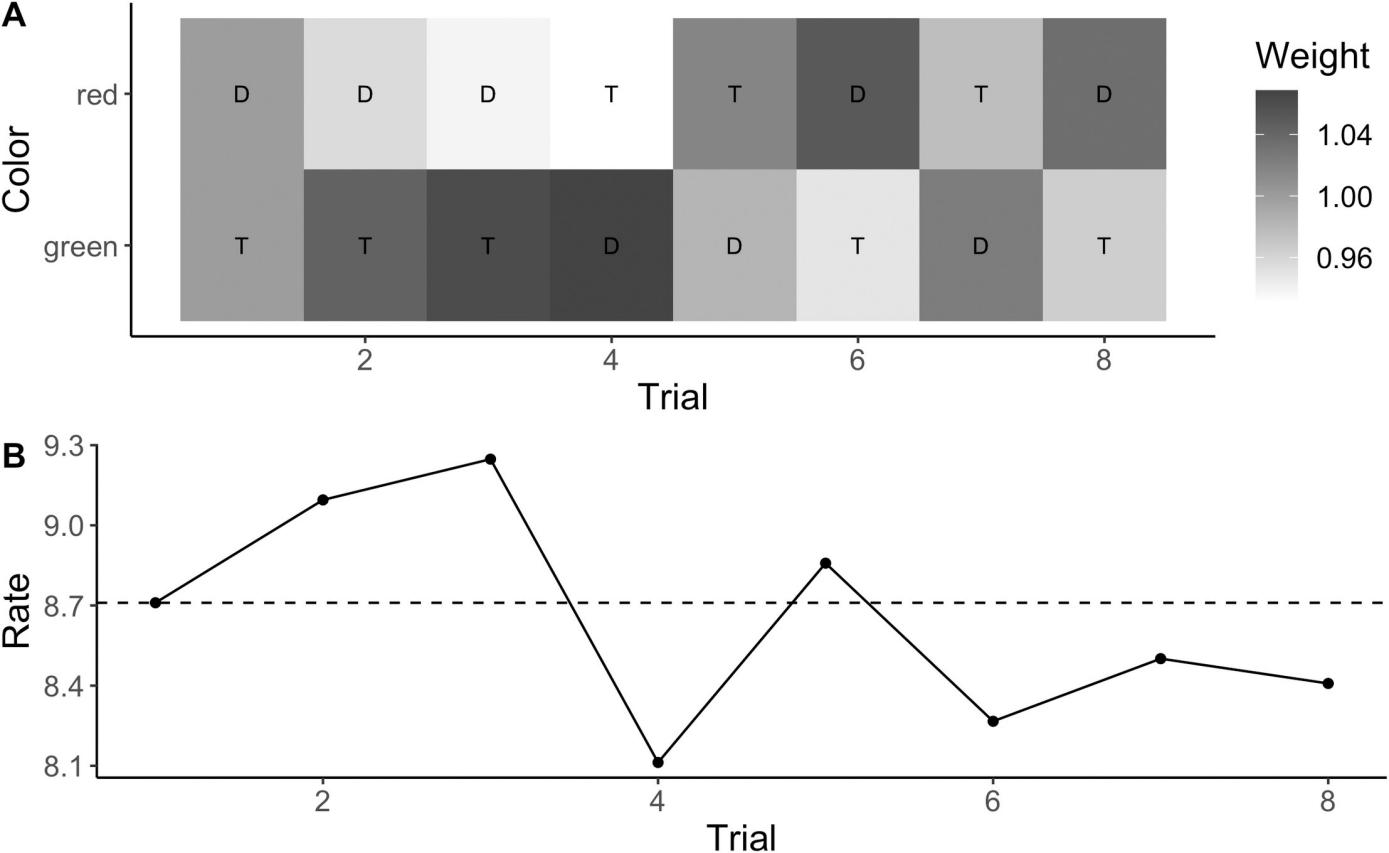
Example of color-based updating. Example of the weight changes to the different colors predicted by the weighted-rate updating rule for the first eight trials of a typical participant (A), and the associated changes in the evidence accumulation rate on the same eight trials (B). The letters “T” and “D” denote the target color and, respectively, the distractor color on each trial. The dashed line marks the baseline evidence accumulation rate. The evidence accumulation rate on each trial was the baseline rate scaled by the weight associated with the target position on that trial.

**Fig 9:**
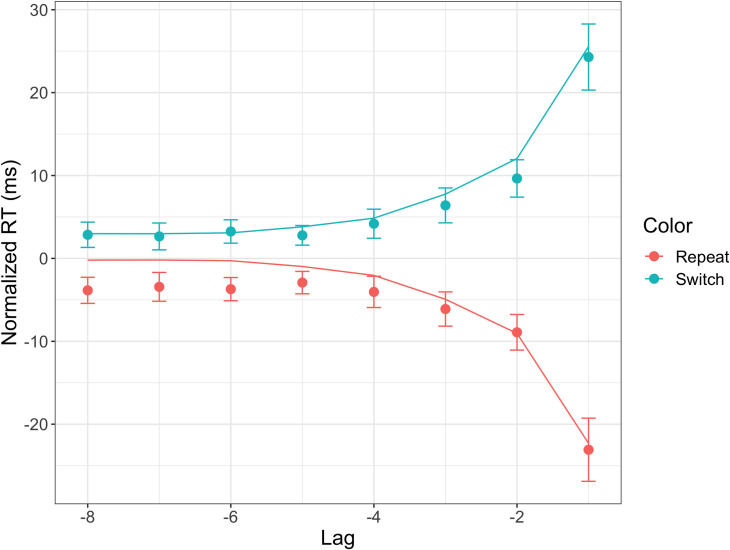
Temporal profile of the color-based inter-trial effects. Mean normalized RT for repeated vs. switched target color on (current) trial *n* compared to (preceding) trial *n-1* (lag 1), *n-2* (lag 2) up to *n-8* (lag 8). Normalized RTs were first averaged across the two sessions for each participant; the resulting (individual mean normalized) RTs were then used to compute the overall means and confidence intervals, across participants. Filled circles depict the behavioral data, lines the model predictions. Error bars represent 95% confidence intervals.

To find the best rule for target color-based updating, we compared the different target color-based updating rules in terms of the mean relative AIC (see Fig 5). The best rule was the position-independent weighted-rate rule (see updating rule 7 in the “Models and updating rules” subsection of the Methods and models section for details). This updating rule assumes that the evidence accumulation rate depends on how much of a limited “weight” resource, which is distributed across both of the possible target colors, is allocated to the target color. This weight is updated after each trial by shifting some weight to the target color on that trial, with partial “forgetting” of old updates (see Fig 8 for an example, Fig 9 for the predicted temporal profile with this updating rule, and Methods and models section for more details).

#### Target position-based updating models

**Fig 10:**
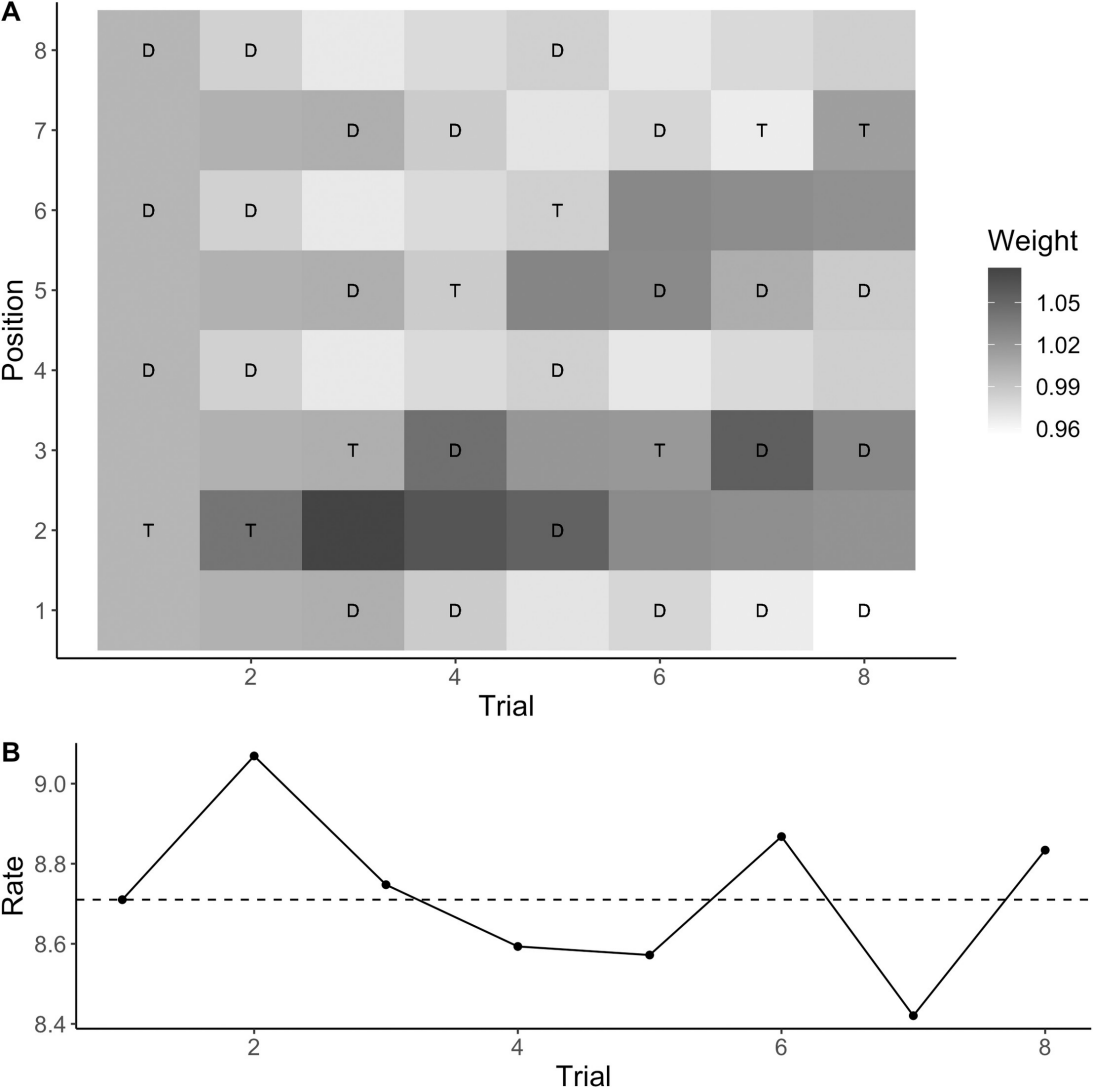
Example of position-based updating. Example of the weight changes to the different positions predicted by the “weighted rate with distractor inhibition” updating rule for the first eight trials of a typical participant (A), and the associated changes in the evidence accumulation rate on the same eight trials (B). The letters “T” and “D” denote the target position and, respectively, the positions of the three distractors on each trial. The dashed line marks the baseline evidence accumulation rate. The evidence accumulation rate on each trial was the baseline rate scaled by the weight associated with the target position on that trial.

**Fig 11:**
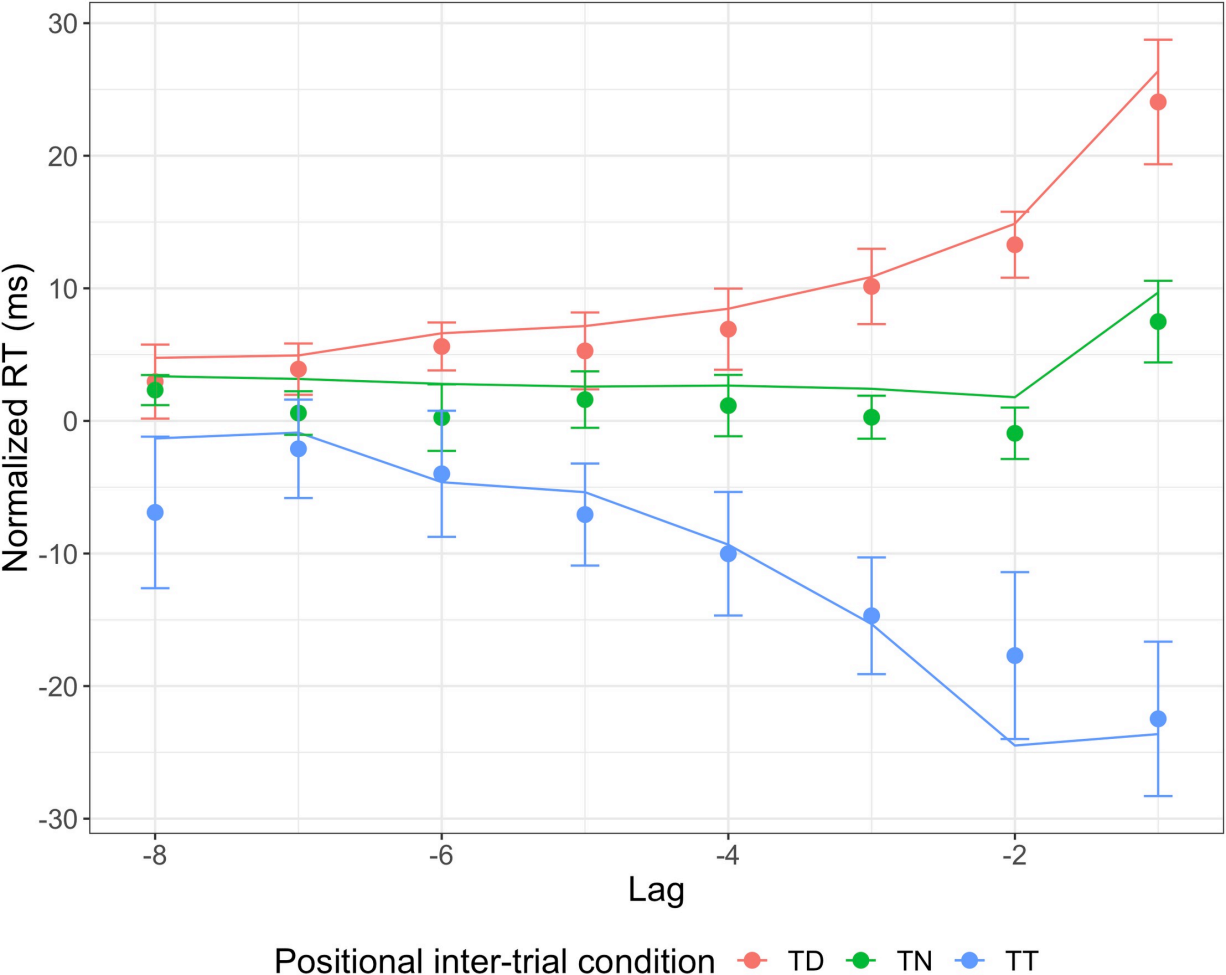
Temporal profile of the position based inter-trial effects. Mean normalized RT for different positional inter-trial conditions, target in previous target condition (TT), target in previous distractor position (TD), target in previously neutral (unoccupied) position (TN), compared to trial n-1 (lag 1), n-2 (lag 2) up to n-8 (lag 8). Normalized RTs were first averaged across the two sessions for each participant; the resulting (individual mean normalized) RTs were then used to compute the overall means and confidence intervals, across participants. Filled circles show the behavioral data, while lines show model predictions. Error bars represent 95% confidence intervals.

Next, we compared the mean relative AIC of position-based updating rules (see Fig 6). The best rule for position-based updating was the weighted-rate with distractor inhibition rule (see updating rule 8 in the “Models and updating rules” subsection of the Methods and models section for details). This updating rule assumes that the evidence accumulation rate depends on how much of a limited weight resource, which is distributed across all the possible target positions, is allocated to the target position. This weight is updated after each trial by shifting weight to the target position on that trial from all other positions, both distractor positions and empty positions, and shifting weight away from the distractor positions on that trial to all other positions, both target positions and empty positions, with partial forgetting of old updates (see Fig 10 for an example and Fig 11 for the predicted temporal profile with this updating rule, and Methods and models section for more details).

#### RCF-based updating models

**Fig 12:**
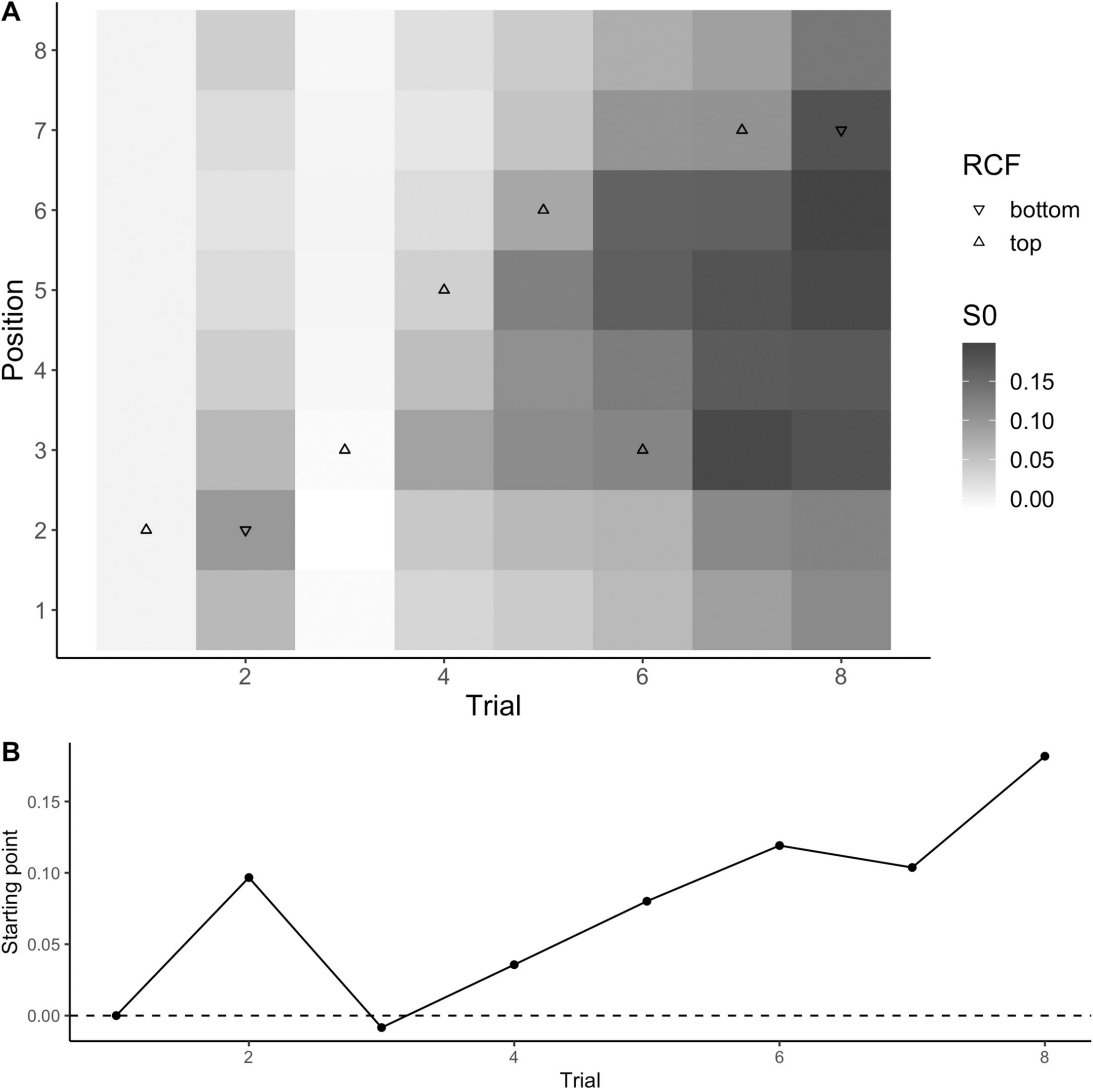
Example of response-based updating. Example of the starting point changes associated with the different positions predicted by the gradient-like dependence on the change of position (i.e., “PG Bayesian S0” updating rule) for the first eight trials of a typical participant (A), and the starting point for the target position on the same eight trials (B). The position of the triangles represent the target position on each trial, and the shape of the triangle indicates the response-critical feature (RCF, i.e., whether the notch was on the top or bottom of the target item).

**Fig 13:**
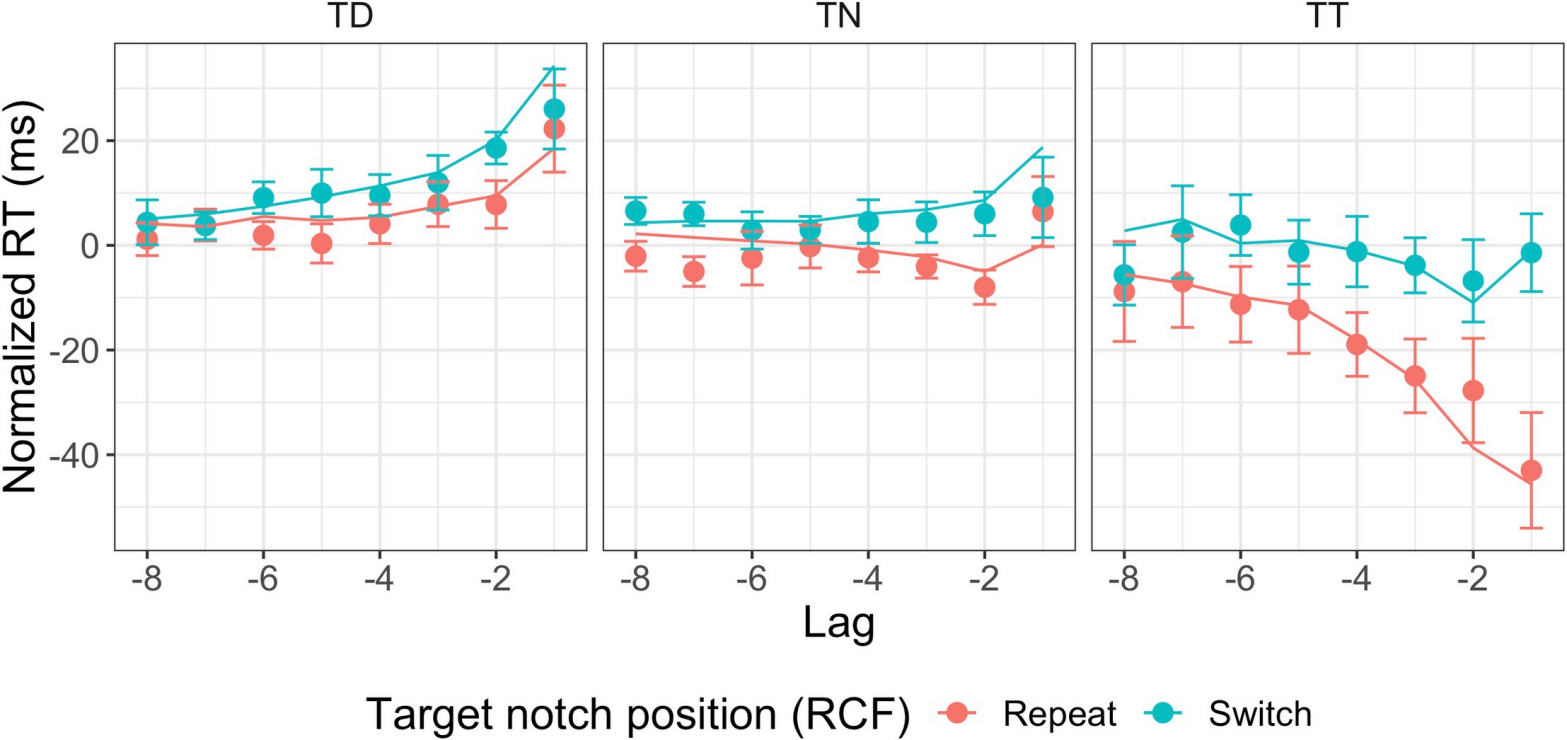
Temporal profile of the response feature-based inter-trial effects. Mean normalized RT for repetition vs. switch of the response defining target feature (notch on top or bottom of the diamond shape), compared to trial n-1 (lag 1), n-2 (lag 2) up to n-8 (lag 8) for the different positional inter-trial conditions, target in previous target condition (TT), target in previous distractor position (TD), target in previously neutral (unoccupied) position (TN). Normalized RTs were first averaged across the two sessions for each participant; the resulting (individual mean normalized) RTs were then used to compute the overall means and confidence intervals, across participants. Filled circles show the behavioral data, while lines show model predictions. Error bars represent 95% confidence intervals. RCF: response-critical feature.

Finally, we compared the mean relative AIC of response-based updating rules (see Fig 7). The best rule was “Position Gradient (PG) Bayesian S0” (see Fig 12 for an example and Fig 13 for the predicted temporal profile and updating rule 4 in the “Models and updating rules” subsection of the Methods and models section for details). This updating rule learns a different starting point for each possible target position, updating the starting point primarily for the actual position where the target occurred, though with some updating carried over to other positions, with the magnitude of the update decreasing with distance (we refer to this a gradient-like dependence on the change of position). Interestingly, both the positionally non-specific version of the Bayesian starting point updating rule and the completely position-specific version (which updates only a single position with no effect on other positions) performed considerably worse.

#### Cross-validation

In order to examine to what extent the better fit of the best updating rules compared to the ‘No-update’ rule was a result of overfitting, we performed cross-validation on the best model and the no-update model. Specifically, since each session consisted of eight blocks of trials, we performed an eight-fold cross-validation, that is: for each block, we evaluated the model’s prediction in that (‘test’) block after training the model on the remaining seven (‘training’) blocks (the stimulus sequence in the test block was still used for updating, so that the starting point and weight would start with the correct values at the beginning of the next block, but participants’ performance in that block was not used for optimizing the model parameters). Table 1 presents the average log-likelihood (logarithm of the likelihood) for the best model as well as the model with no updating, each evaluated on the training set (averaging the log-likelihood in each block across the seven folds in which that block was included in the training set) as well as on the test set. The average log-likelihood was somewhat worse when evaluated on the test set, indicative of some degree of overfitting, and this difference was larger for the best model compared to the no-update model, suggesting there was some overfitting of the updating rules, in addition to the evidence accumulation model. Importantly, however, even when evaluated on the test set, the log-likelihood was substantially better for the best model compared to the no-update model, indicating that the updating rules did capture patterns in the data that generalize across blocks. This conclusion is further supported by the cross-validated out-of-fold predictions for the temporal profiles of the inter-trial effects presented in S6 Appendix, which fit the pattern in the data nearly as well as the corresponding predictions without cross-validation (depicted in Figs 9, 11 and 13 below).

**Table 1:** Average log-likelihood, across participants and sessions, for the best model and the no-update model, evaluated on the training set as well as on the test set, and the difference between the test and training set log-likelihood (right column). The bottom column shows the difference in log-likelihood between the best model and the no-update model.

	Training set	Test set	Difference (test—train)
No-update model log-likelihood	574	550	-24
Best model log-likelihood	639	585	-54
Δlog-likelihood	65	35	-30

### Temporal profiles of the inter-trial effects and model predictions

Having seen which updating rule best accounts for each type of inter-trial effect, in this section we will explore what prediction those updating rules make for the temporal profile of each inter-trial effect and how well this matches the temporal profiles in the experimental data. In the next three subsections, we explore how well the model framework predicts the three different types of inter-trial effect: (i) inter-trial effects related to repetition/switch of the target-defining color, more precisely: repetition of both the target and distractor colors versus swapping of the target and distractor colors; (ii) inter-trial effects related to the target position, for which there are three different transition conditions: target at previous target position, target at previously unoccupied (neutral) position, and target at previous distractor position; (iii) inter-trial effects related to the response-critical feature, the position (top or bottom) of the notch in the target item. All figures in the next three sections show normalized RTs, where the overall mean RT has been subtracted from the mean in each condition, for each participant and session (i.e., a positive normalized RT means that the response time is slower than overall average in a particular condition, while a negative normalized RT means it is faster than overall average). To make the model predictions directly comparable to the behavioral data, the mean RTs (from the behavioral data) were also subtracted from the model predictions. All model predictions are based on the best model, that is, using the factor level that resulted in the lowest AIC for each of the factors (see the model comparison section above); accordingly, the relevant figures below (Figs 9, 11 and 13) are all based on the exact same model (see S2 Appendix for the parameters of the model fits), applying all of the winning updating rules (even though each of these figures only illustrates the effects of one, or two in the case of Fig 13, of these rules).

#### Color-based inter-trial effects

Fig 8 illustrates the predictions of the best updating rule for color-based inter-trial effects, that is, the “weighted-rate” rule, for the first eight trials of a typical participant. The two colors start out receiving equal weight (each with a weight of 1 so that the rate for each color is the baseline rate), and after each trial some weight is shifted to the target color on that trial, while additionally there is partial forgetting so that the weights are partially reset to their starting values (see modeling part of Methods and models section for details). The figure illustrates how repetition benefits arise in the model: On trials 2, 3, and 5, the same target color is repeated as on the previous trial; on these trials, the weight associated with the target color is larger than one because some weight has just been shifted to that color; on trials 4 and 6–8, by contrast, the target color has changed from the previous trial, resulting in weight of less than one associated with the target color because some weight was just shifted away from that color. The figure also illustrates influences from more than one trial back: the weight associated with the target color is larger on trial 3 compared to trial 2, because this is the second repetition in a row and the weight shifts accumulate; by contrast, the weight is considerably lower on trial 5 because this color repetition was preceded by a sequence of three trials with the other target color.

The color-based inter-trial effects were predicted well by this updating rule (shown in Fig 9). Response times are faster (by 56 ms, on average) when the color from trial *n-1* is repeated on trial *n*. The same pattern is found for the repetition versus switch of the color from more than one trial back, although the magnitude of the effect decreases with increasing lag. This pattern is captured well by the model, although the model somewhat underestimates the effect for longer lags (i.e., for influences from four or more trials back), while it, if anything, slightly overestimates the effect at the shortest lags (influences from one to three trials back).

#### Position-based inter-trial effects

The best updating rule for target position-based inter-trial effects (illustrated in Fig 10), that is, the “weighted rate with distractor inhibition” rule, followed a similar logic to that for the color-based effects. All eight positions start with equal weight (each with a weight of 1 so that the rate for each position is the baseline rate), and after each trial some weight is shifted to the target position on that trial from all other positions (i.e., both distractor positions and unoccupied positions), and away from the distractor positions on that trial to all other positions (i.e., both the target position and unoccupied positions; see modeling part of Methods and models section for details). This results in increased weight associated with the previous target position and decreased weight associated with the previous distractor positions, while the weight associated with the neutral positions does not change much because they both lose weight to the target position and receive weight from the distractor positions. This explains why the model predicts facilitation of response times for “target at previous target position” transitions (TT) and a cost for “target to previous distractor position” transitions (TD) compared to when the target moves to a previously neutral (empty) position (TN). The figure also illustrates influences from more than one trial back: both trial 4 and trial 7 are TD transitions, but the weight associated with the target position is much lower on trial 7, because the distractors had appeared at that position an additional two times in the recent trial history.

The model also predicts well for the position-based inter-trial effects (Fig 11). Compared to when the target on trial *n* appeared at a position which was neutral (unoccupied) on trial n-1 (TN), RTs were faster (by 36 ms, on average) when the target appeared at the previous target position (TT), and slower (by 17 ms) when the target appeared at a previous distractor position. The same pattern of positional inter-trial effects is seen for transitions from more than one trial back, though the size of the effect decreased with increasing lag. The model predicts this pattern, although it predicts a slightly larger target-target transition benefit relative to the distractor-target transition cost compared to the behavioral data.

#### Response-based inter-trial effects

The best updating rule for the response-based inter-trial effects: “PG Bayesian S0” (illustrated in Fig 12) assumed that participants learned a separate response bias (starting point of the evidence accumulation model) for each position, based on the frequency with which each response has recently been associated with that position, but that updating of the bias in the current target position on any trial partially carried over to other positions in a gradient-like way. In the example shown in Fig 12, the starting point is biased towards the boundary associated with a “notch on top” response after the first trial, where the strongest bias is associated with the position where the target occurred but with some bias also for other positions. On the second trial, the target position was repeated but the notch position changed–so the bias learned from the first trial is nearly cancelled. However, because of the memory decay, the new notch position had a somewhat stronger influence than the old one; so, instead of complete cancellation, the result was a small bias in the other direction. The next five trials all had the notch on the top, but with the target occuring in different positions, resulting in a build-up of a bias towards that response associated with all positions.

Because inter-trial effects for the response primarily occurred when the target position was repeated (see Fig 3), the RCF-based inter-trial effects are shown separately for the different positional inter-trial transition conditions (Fig 13). The model predicts well in the repeated target position condition (TT). However, for the other conditions, the model slightly overestimates the inter-trial effects from one trial back and somewhat underestimates those for longer lags. This happens because in the behavioral data, there are virtually no effects of repetition/switch of the RCF from a single trial back when the target position does not repeat, while there are some such effects from two and more trials back.

## Discussion

In the present study, we applied the evidence accumulation framework that we had previously used to model inter-trial effects in pop-out visual search tasks [12] to the data from a “priming of pop-out” study [19]. The primary aims were to examine, through modeling, the mechanisms underlying the temporal profile of *n*-back inter-trial effects on mean RTs and to investigate the degree of spatial specificity of the inter-trial effects for the response-critical target feature and the target-defining feature. Comparing 1920 different models (each possible combination of 2 evidence accumulation rules, 12 updating rules for the response, 8 updating rules for color and 10 for position) for each of 14 participants, we showed that the best models in general predicted the temporal profiles of the inter-trial effects well (see Figs 9, 11 and 13), with some interesting deviations that are discussed in more detail below.

### Feature-, position-, and response-based inter-trial dynamics in PoP

Model comparisons suggest that the best model to predict the inter-trial effects related to the response-critical feature (RCF, i.e., notch position) is to update the starting point of the evidence accumulation process. This is consistent with what we found in our previous study [12], where a factorial model comparison revealed that in three pop-out visual search experiments, inter-trial effects for the RCF were best captured by updating of the starting point, both for a simple detection task (requiring a target-present/-absent response) and a target discrimination task (in which participants responded to the target-defining dimension, color/orientation). Interestingly, for the priming pop-out task, the best updating rule was the version of “position gradient” updating, which learns a different starting point for each target position, but updates multiple positions after each trial, with the magnitude of the update decreasing with distance from the target position. This performed better than either a completely position-independent rule which learns a single starting point for all positions, or a completely position-specific rule which learns a different starting point for each position and updates only the target position after each trial. Based on an analysis of inter-trial effects from a single trial back, the superiority of the “gradient” rule over the completely position-specific one may seem surprising, given that there was no evidence of any inter-trial effects related to the RCF except when the target position was repeated (see Fig 3). However, an analysis of the effect of the further-back history of the RCF and target position (see Fig 13) reveals the reason for the superiority of the “gradient” rule: repetition/switch of the RCF from two trials back or more (trial *m*<*n*-1) influences response times on trial *n* even when the target position was different (on trial *m* compared to trial *n*). This ‘long-term’ aspect of the inter-trial effects–the fact that RCF-based effects from trial *n*-1 are almost completely position-specific, but effects from trial *m*<n-1 are less position-specific–was not adequately captured by our best updating rule. One possibility is that it reflects an episodic retrieval mechanism [4,36], that is, the response from the previous trial has been bound into a memory of the response episode together with the position from the previous trial (an ‘event file’), so that when the same position is repeated the previous response is automatically retrieved, resulting in faster responses when both the position and the response are repeated. The reduced position specificity for inter-trial effects from more than one trial back could then be a result of partial forgetting, that is, for episodes retrieved from a single trial back the position is remembered with sufficient accuracy to make the effect fully position-specific, whereas for older episodes the quality of the memory of the position has deteriorated, causing the influence from that trial to spread to other positions. However, our “position spreading” updating rule, which was designed to investigate this possibility, while it qualitatively predicted the same pattern of inter-trial effects seen in the data (i.e. highly position specific inter-trial effects from one trial back, but less position specific for more than one trial back, see S3 Appendix), actually performed somewhat worse than the “position gradient” rule in terms of AIC. This could mean that the pattern of inter-trial effects does not reflect an initially position-specific memory episode which later spreads; instead, it could also be that our “position spreading” updating rule did not implement the spreading in the right way, as there are many different ways in which position-specific learning followed by spreading might potentially work.

For the feature-related priming of pop-out effect [3], the best updating rule used updating of the evidence accumulation rate, based on, after each trial, shifting some of a shared “weight” resource to the target color on that trial. In our previous study, a very similar “weighted-rate” rule best explained inter-trial effects for the target dimension [12]. The small deviations of the model fit from the behavioral data consisted in the model slightly underestimating the influence of older trial history (older than trial *n*-3). This deviation might possibly reflect a separate updating mechanism, operating on a slower time scale, with slower memory decay. Priming of pop-out with different speeds of memory decay has previously been proposed based on experiments in which sustained repetition of one target color was followed by fewer trials with a different target color [37]. This resulted in initially stronger priming for the second, more recently primed color, followed by a period of better performance for the first color, that is, the longer build-up period resulted in slower memory decay. The possibility that there may be different priming mechanisms with different speeds of memory decay is also supported by a modeling study showing that the temporal kernel for priming of the target-defining feature, capturing how the priming effect decays with time, is better fitted by a sum of two exponential functions, one with slower and one with faster decay, than a single exponential function [21].

The target position also plays an important role in inter-trial priming, as known as to the positional priming of pop-out [8,19]. Our model comparisons suggested the best updating rule, similar to the updating rule for the feature-related priming of pop-out, used updating of the evidence accumulation rate, based on, after each trial, shifting some of a shared “weight” resource to the target position and away from the distractor positions on that trial. The second best alternative updating rule, which is close to the best one, for the positional priming of pop-out is to update the component of the non-decision time in the evidence accumulation process based on the target position repetition and switch. A further analysis (see S1 Appendix) based on counting how often an updating rule appeared in the best model for an individual participant and session, showed that 71% of the best model fits for the position-based updating used an updating rule operating on the rate while 29% used an updating rule operating on the non-decision time (compared to 96% on rate and 4% on non-decision time for color and 82% on starting point and 11% on non-decision time for the RCF). In most cases, the second best position-based updating rule operated on the same parameter as the best rule (70% for the participants for who the best rule operated on the rate, and 100% for the participants for who the best rule operated on the NDT) but using a different updating rule (in most cases either weighted rate/NDT without distractor inhibition or with matched distractor inhibition). This relatively high proportion of the best models using a rule based on non-decision time combined with the relatively small difference in AIC between the best non-decision time updating rule and the overall best rule suggests that there may be more than one mechanism underlying position-based inter-trial effects. If the response related inter-trial effects reflect an episodic retrieval mechanism as discussed above, then one possibility is that, in addition to the response itself, the S-R mapping has also been bound into the same event file with the position, and the second mechanism for position-based inter-trial effects (in addition to the attentional “weighting” mechanism discussed above) could involve faster retrieval of the S-R mapping when the target occurs at the same position. The reason why there was much less evidence of any second mechanism for color-based inter-trial effects could then be that there was less strong binding of the color, compared to the position, and response into the episode, which is also supported by the weaker interaction between color repetition and response repetition compared to position repetition and response repetition in the mean RTs. This might possibly be a consequence of the singleton detection nature of the task, that is: participants were searching for a color singleton, which could be either of two colors both of which were also used as distractor colors, rather than an item of a specific color, perhaps making them less likely to include the specific color in the event file.

Taken together, the best model we found used updating of the starting point based on the history of the responses and updating of the evidence accumulation rate based on the history of the color and target position. This suggests that the response-critical feature repetition/switch induces a response bias in the direction of recent responses given to (a stimulus at) the exact same or a nearby position. By contrast, the target color repetition/switch alters the efficiency of processing of the target. Similarly, inter-trial effects for position may reflect more efficient processing of a recent target location and less efficient processing of recent distractor positions. In both cases (target color and position), more attentional “weight” resources are allocated to recent target properties. Of note, the present model comparison for the feature priming of pop-out could not distinguish between effects of resources being moved to the target color and effects of resources being moved away from the distractor color, owing to the design of the present experiment. In the behavioral study, as has been common practice in experiments on priming of pop-out (e.g. [3,8]), the target color and distractor color were either both repeated or they were swapped, so there was no condition in which only the target color, but not the distractor color, was repeated or vice versa. However, previous experiments that have examined the separate effects of repetition/switch of the target and the distractor color have found repetition benefits for both, although of unequal size (e.g., [38]; Experiment 8 in [3,39]).

For all updating comparisons, both the LATER and the DDM models made a consistent prediction for the best updating rules, though, the LATER models outperformed the DDMs in terms of AICs across. This was also the case in a previous study with the pop-out search task [12]. One modification we did for the LATER model is that we included a non-decision time to make it comparable to the DDM. This additional parameter may contribute in some degree for the flexible fitting with the LATER. In addition, it should be noted that we used a closed-form approximation of the RT distribution predicted by the DDM [40] to keep the computational demands at a manageable level. This approximation may not capture trial-to-trial variability of the non-decision time, which might also contribute to the difference in AIC between the LATER model and the DDM. Nevertheless, the most critical outcome from both models is that the best updating rules were consistent.

It should be noted that here we used one single stage of evidence accumulation process, while in reality each trial may have involved multiple stages of perceptual decisions. Even if the target is the first item to be attended to, observers would need to both confirm that the item is the target and also make a perceptual decision about the RCF (notch position). In addition, the target is likely not to be the first item selected on a significant proportion of trials [16]. Therefore, the model parameters, starting point, drift rate and non-decision time have to be interpreted with some caution. However, the temporal profile of inter-trial effects is likely to depend more on the memory/forgetting mechanisms of the different updating rules than on the details of the model of perceptual decisions on an individual trial, which would explain why our simple model could still capture these temporal profiles quite well.

Of note, although the present modeling approach inherently assumes a one-stage architecture of decision making, the results can be reconciled with current notions of the functional architecture of pop-out visual search in so-called ‘compound’ tasks, which require a response to a target feature (here: position of the notch in the target shape: top vs. bottom) that is separate from the detection-critical feature (here: odd-one-out color of the target shape). Current notions, in the Guided Search framework (e.g., [41–43]), envisage a two-stage architecture with a preattentive stage determining which locations to select for focal-attentional processing, followed by a post-selective stage of attentional analysis of the selected items. Attentional selection is based on the computation of ‘priority’ signals which themselves are ‘feature-blind’: they signal that an item (at a high-priority location) is maximally different from the surrounding items (‘feature contrast’), but not in what feature(s) it differs from these items. The latter is established in post-selective processing, which determines whether the item in the focus of attention is actually a target (rather than a non-target) and extracts any further features necessary for choosing the required response (‘stimulus-response mapping’). Even though couched in terms of a one-stage decision process, the present findings appear to be compatible with this two-stage architecture:

(i) The fact that both target color- and position-based intertrial history affects the rate of evidence accumulation is consistent with models of search guidance based on the computation of a map of attentional priority signals: in a parallel-competitive process, that location is selected for attentional processing (extraction of stimulus and response-critical features) for which the priority signal crosses the attention-triggering threshold first. Within such a framework, color history (top-down) modulates bottom-up feature (contrast) coding, with recent target colors being up-weighted and distractor colors correspondingly down-weighted in a manner that operates equally and in parallel across the respective feature map, giving rise to a spatially non-specific feature repetition effect; and position history modulates the landscape of the priority map via intra-map connections, with recent target locations being up-weighted and distractor locations correspondingly down-weighted, giving rise to featurally non-specific position repetition effects (e.g., [7,44]). Evidence that the combined dynamics is mediated via separable (featural and positional) weighting mechanisms (modulating the feedforward connections from the feature maps to the priority map and, respectively, the intra-map connections within the priority map) may also be gleaned from the fact that the decay factors differ between the two types of repetitions effects. This is further supported by a reanalysis of Gokce et al.’s [19], EEG data, which revealed that the (event-related) N2pc component is modulated by both color- and position-based inter-trial transitions (see S5 Appendix): Repeating the same target color resulted in a shorter peak latency of the N2pc, compared to switching the colors. And positional inter-trial effects were expressed in the amplitude, rather than the timing, of the N2pc component: compared to targets at previously neutral locations (TN condition), the amplitude was smaller in the repeated target-position (TT) condition and larger in the target-at-previous-distractor-location (TD) condition. This could mean that, while the attentional priority signal emerged as fast at a previous distractor as at a previous target location (i.e., ‘attentional shifting was equally rapid), more ‘effort’ needed to be expended to ‘attentionally engage’ a prioritized item at a previous distractor location–consistent with, for example, [45]. In any case, these findings indicate that both color and position repetitions facilitate search at an early, attentional-selection stage.

And (ii) the fact that the effect of repetition of the response-critical feature is tied to the target location (i.e., it occurs only for TT transitions) would support accounts according to which this effect arises at a post-selective (focal-attentional) stage which implements, and buffers, episodic bindings between target features (including its position) and the response selected based on these features. See, for instance, Hommel et al.’s [46] episodic ‘theory of event coding’, or TEC, which was designed to explain ‘partial repetition effects’, such as prolonged RTs to a target which occurs at the same location as that on the previous trial, but which requires a changed response: the idea is that the position repetition re-activates the S-R binding (‘event file’) established on the previous trial, invoking a bias towards giving the same response; this gives rise to fast reactions when the response (or the response critical feature) repeats, but to slowed reactions when there is a change (because in this case the induced bias needs to be overcome; see also episodic-retrieval account of Huang et al. [4] and the more general ‘Binding and Retrieval in Action Control (BRAC)’ framework recently proposed by Frings et al. [36]). These notions are consistent with the present findings that RCF repetitions influence the starting point of the evidence accumulation process in terms of a (repetition) bias–and that they do so exclusively for target position repetitions, because episodic (S-R) event files are set up only for attentionally selected and analyzed ‘target’ stimuli. Note that we did not model the possibility that response-repetition effects might also be dependent on feature repetition (i.e., that the target-defining feature may also be part of the retrieved event), because the BF did not provide conclusive evidence for an interaction between response repetition and feature repetition. However, this may be due to lower uncertainty for target color (2 alternatives) than for target position (8 alternatives) in our paradigm. Recent evidence suggests that reliance on retrieval depends on uncertainty (e.g., [36,47]).

Of course, to what extent our one-stage decision models can completely capture what is essentially a two-stage dynamic process needs to be seen in future modeling work, perhaps bringing together models of tasks that require pre-attentive target selection with no (or minimal) demands on post-selective processing (such as simple-detection tasks) and tasks that require post-selective processing with no (or minimal) target selection (e.g., presenting targets at validly pre-cued locations, as in [16]). However, S4 Appendix can ascertain that our single-stage model captures much of the dynamics. In this appendix, we show that a multi-stage model, with separate item-selection, target-confirmation, and response-selection stages, makes very similar predictions for the respective temporal inter-trial effect profiles when the winning position- and color-based updating rules are applied to the rate parameter of the item-selection stage and the winning response-based updating rule is applied to the starting point of the response-selection stage. We take this to indicate that our single-stage model provides a sufficiently good approximation for the purpose of determining which updating rules best explain the temporal profiles of the inter-trial effects.

### Relation between featural and dimensional ‘priming’ in visual singleton search

From a theoretical perspective, one final issue to be discussed concerns the relations between target feature priming (PoP), which is characterized by long-lasting inter-trial effects, and dimension priming (e.g., [5]), which is characterized by relatively short-lived inter-trial effects (modeled in our previous paper [12]). We believe that, due to the ‘nature’ of the displays typically used in the two paradigms (PoP: three or four regularly arranged items that are relatively widely spaced, with the target being defined in a fixed dimension; dimension priming: multi-item arrays of densely arranged items, maximizing local target feature contrast, with the target-defining dimension being variable), dimension priming impacts the computation of target saliency (which is thought to be dimension-specific in nature; see also [48]), with minimal involvement of top-down feature-based processes (see, e.g., [49,50]). Specifically, our dimension-weighting account (e.g., [5,6]) assumes that dimensional priming is implemented in terms of the competitive up-/down-modulation of the weights of feature contrast signals computed within the alternative, potentially target-defining dimensions in their transfer to the supra-dimensional ‘priority’ map, which then drives the allocation of attention. In contrast, given that the target is relatively non-salient in typical PoP paradigms (resulting in failure to pop-out on a significant number of trials; e.g., [16,17]), feature-based top-down biasing (along with positional cues as well as ‘Gestalt’-based processes; regarding the latter, see [13]) comes prominently into play in PoP paradigms.

Thus, in typical PoP paradigms with color-defined targets, observers would have to positively establish which color the target is in order to be certain that the response is not made to a distractor item. This would involve matching of a selected item to a ‘target template’ held in working-memory (WM). If a match is established, the activation of the template is increased (and perhaps that of the alternative template decreased, generating inter-trial effects at this post-selective stage; see below). Thus, given that the template in turn biases early, entry-level feature coding (as demonstrated in a plethora of studies, e.g.,[42,51]), the relative activation state of the templates determines the ‘priming’ of the respective feature detectors, and thus the computation of feature contrast within the (target-defining) color dimension. This in turn would speed up the computation of priority for the target item, albeit unreliably. Given noise in the contrast-computation process and given that the ‘wrong’ feature template might boost distractor feature coding, it can happen that a distractor achieves a higher contrast than the target (especially on a trial with a color swap), leading to selection of a non-target item. By comparison, in dimension-priming paradigms, the target feature contrast is so high that the wrong item gets virtually never selected; and observers can respond reliably “target-present” based on the presence of a feature-contrast or ‘priority’ signal–without needing to know exactly what feature defines the target (see, e.g., [52]), that is: the demands on target-template matching are minimal, minimizing the biasing of entry-level target feature coding.

Thus, in both paradigms, ‘prediction’ of the target feature (PoP) or target dimension (dimension priming) would be expressed in a speeding (of the rate of evidence accumulation) at the pre-attentive, target-selection stage (as evidenced by ‘early’ attention-related N2pc effects in both paradigms: e.g, PoP: [19]; see also current S5 Appendix); dimension priming: [53,54]). Positional inter-trial effects are seen also with both paradigms (dimension priming: [53,54]) and produce N2pc effects (at least in PoP; see [19] and S5 Appendix). These effects would be consistent with faster accumulation of selection-related, ‘priority’ evidence in both paradigms.

In addition, post-selective target-checking might be a contributory factor, though predominantly in PoP (where this stage becomes necessary for the reasons discussed above): assuming that some template is in a higher state of activation (because it matched the target on the previous trial), again a match of the current target to that template might be expedited. Behavioral evidence suggests that template checking operates in a more serial fashion (compared to the parallel co-active computation of selection priority), that is, a selected item is checked only against one template at the time (see [55]). Assuming that the winning template is carried over to the next trial (i.e., the selected item is first matched against this template), this could give rise to feature- (and dimension-) priming effects at this level. Dedicated modeling work would be required to ascertain whether this would be expressed in a shift of the starting point or the rate of evidence accumulation at this post-selective stage.

In summary, we believe that both feature-priming and dimension-priming effects can be accommodated within the same functional architecture of search guidance, such as the early level of feature extraction (priming of feature detectors) and, respectively, the integration (summation) of feature-contrast signals across dimensions by the priority map (modulating the integration weights of dimensional signals, giving rise dimension priming). The fact that the two types of priming have differential time courses (long n-back effects for feature priming, relatively short-lived effects for dimension priming) would be consistent with the idea that the two types of priming arise at different levels in the search architecture. In particular, we believe that feature priming involves some interaction between target templates held in WM and early feature coding, so that the inter-trial effects could arise at one or the other or both levels.

## Conclusion

In conclusion, we found that our modeling framework, consisting of an evidence accumulation model with trial-to-trial updating of the parameters based on stimulus history, can in general accurately predict the pattern of RT variability resulting from priming from one to eight trials back. The modeling results suggested that the RT benefits resulting from repetition of the color or position of the target can be best understood as short-term memory based up-modulations of the target’s perceptual priority (in the model captured by re-distribution of a weight resource which modulates the evidence accumulation rate), while the benefits resulting from repetitions of the response may instead reflect a bias towards the previous response (in the model captured by a shift of the starting point), perhaps resulting from retrieval of an an event file created on the previous trial. Interestingly, while the response based priming appears to be nearly completely position-specific for priming from a single trial back (i.e., there was only a response repetition benefit if the position was also repeated), it seems to be less position-specific for priming from more than one trial back, which could reflect forgetting of the precise position in older event files.

## Methods and models

### Ethics statement

Written informed consent was obtained prior to the start of the experiment and anonymity of observers’ data was guaranteed. The study was approved by the LMU Department of Psychology Ethics Committee.

The behavioral data modeled here come from a previously published, combined behavioral and EEG study [19]. While we describe the most important behavioral methods below, more detail can be obtained from the original publication [19].

### Participants

14 participants (8 female, mean age: 23, SD: 1.74 years), recruited from the participant panel of the unit of Experimental Psychology, LMU Munich, took part in the study. All participants had normal or corrected-to-normal visual acuity, and all reported normal color vision and being right-handed. Participants were naïve as to the purpose of the study. Participants were paid at a rate of 8 € per hour, or received course credits for their participation.

### Apparatus and stimuli

The search display consisted of four items, appearing in four out of eight possible stimulus locations arranged equidistantly on a circular layout. The four items could appear in either a diamond configuration, with items appearing on the top, bottom, leftmost and rightmost positions of the circle, or a square configuration. Each item was a diamond shape with a section (“notch” on either the top or the bottom cut-off. The items were either red or green and one of the four items, the target, was always differently colored from the other three (see Fig 2).

### Procedure

The experiment was divided into two sections each consisting of eight blocks of 112 trials. Each trial started with a fixation cross presented for 500 ms, followed by the stimulus display presented for 200 ms. The trial then ended when the participant made a response or after one second if the participant had not yet responded. The mapping between the notch positions and the response buttons in the first session of the experiment was counterbalanced across participants and it was reversed in the second session. On trials where no response was given within one second and on trials with an incorrect response feedback was given (“Too slow”/”Error”) and presented for 1 s.

### Design and task

The task of the participants was to find the uniquely colored item, the target, and respond with a left or right mouse button press depending on whether the notch on the target was on the top or bottom. The target was red with green distractors and green with red distractors on an equal number of trials. The position of the notch for each item was determined randomly on each trial. The sequence of target positions was chosen in such a way that the target appeared in each of the eight possible positions equally often, while transitions from trial *n*-1 to trial *n* were equally often to the previous target location, a previous distractor location, or a previously empty location (by switching the item configuration between the diamond and square configurations).

### Bayes factors

Bayesian ANOVA and associated post-hoc tests were performed using JASP 0.10 (http://www.jasp-stats.org) with default settings. All Bayes factors for main effects and interactions in the ANOVA are ‘inclusion’ Bayes factors calculated across matched models. Inclusion Bayes factors compare models with a particular predictor to models that exclude that predictor. That is, they indicate the amount of change from prior inclusion odds (i.e., the ratio between the total prior probability for models including a predictor and the prior probability for models that do not include it) to posterior inclusion odds. We used inclusion Bayes factors calculated across matched models meaning that models that contain higher order interactions involving the predictor of interest were excluded from the set of models on which the total prior and posterior odds were based. Inclusion Bayes factors provide a measure of the extent to which the data support inclusion of a factor in the model. Bayesian t-tests were performed using the ttestBF function of the R package ‘BayesFactor’ with the default setting (rscale = “medium”).

### Models and updating rules

Each model consisted of an evidence accumulation model: either the LATER model or the DDM, and three updating rules, each of which specified how one aspect of stimulus history should affect the trial-to-trial change of a parameter of the evidence accumulation model, or in some cases how two aspects of stimulus history jointly should affect parameter changes (the “position-dependent” and “position-gradient” rules: rules 3–5 and 15–18 below). There was one such updating rule for the response-defining feature, one for the target color, and one for the target position, and in each case one of the factor levels specified that no updating at all should take place. For the DDM, we used a closed-form approximation [43], adding a scaling parameter that determined the size of the random component of the drift diffusion model. This was necessary since our rule for updating the starting point made the scale non-arbitrary. Models were fitted using maximum likelihood, using the R function ‘constrOptim’ to find the minimum value of the negative log likelihood. Error trials and outliers were excluded from the calculation of the likelihood, but were included when implementing the updating rules. Outliers were defined as trials with RTs slower than 1.5 s or faster than 200 ms.

To make sure we found the best possible fit for each combination of updating rules, we used an inner and an outer optimization process. The inner optimization process was run for each combination of parameters that was tested by the outer optimization process, to find the best possible values of the inner parameters for those values of the outer parameters. The inner parameters were the parameters of the evidence accumulation model itself, except for the non-decision time which was an outer parameter. For the LATER model, the inner parameters were the starting point boundary separation, and the mean and standard deviation of the distribution for the rate. For the DDM, the inner parameters were the starting point boundary separation, the rate, and the scaling parameter. The outer parameters were the non-decision time and 0 to 3 parameters for each updating rule (see descriptions of the individual updating rules below for details).

Below we describe the different updating rules. Because many of these could be applied to more than one aspect of stimulus history, namely, target color, target notch position (the response-critical feature or RCF), or target position, we refer to the aspect of stimulus history based on which updating was performed using the generic term “updating variable” (or UV). For color-based updating and RCF-based updating, the UV can take two different values (for color: green target with red distractors or red target with green distractors; for the RCF: notch on top or bottom of the target item). For position-based updating, it could take eight values corresponding to the eight possible target positions.

#### Updating rule 1: No updating

With this updating rule, the updating variable contributed no change of any model parameters from trial to trial. This serves as the baseline.

#### Updating rule 2: Position independent (PI) Bayesian S0

With this updating rule, the starting point (S_0_) was updated based on the history of the updating variable. In line with Gold and Shadlen [26] and Noorani and Carpenter [32], we assume that S_0_ is determined by the logarithm of the prior odds of two decision outcomes (H vs. ~H, the two possible perceptual decisions regarding the value of the updating variable):

$$S_{0}=log\frac{P(H)}{1-P(H)}.$$ (1)

We further assume that the prior probability *P*(*H*), rather than being fixed, is updated trial-wise according to Bayesian inference, because participants are learning the frequencies of different stimulus properties and using this knowledge as a prior when making perceptual decisions. We model this updating using a Beta distribution as the starting distribution on the prior (a hyperprior) and updating this based on the Bayes rule after each trial using the Bernoulli likelihood:

$$P(H_{t}|X_{t\ldots 1})\propto P(X_{t}|H_{t})*P(H_{t}|X_{(t-1)\ldots 1}).$$ (2)

It started out unbiased, i.e., the two parameters of the Beta distribution initially had the same value *β*_0_. In addition to the Bayesian updating, this updating rule also incorporated a forgetting mechanism based on the Dynamic Belief Model (DBM; [35]), such that after each trial there was a probability *α* that the prior was redrawn from the starting distribution P(H_0_):

$$P(H_{t+1}|X_{t\ldots 1})=\alpha P(H_{t}|X_{t\ldots 1})+(1-\alpha )P(H_{0}).$$ (3)

This updating rule contributes two parameters, *α* and *β*_0_, to the model.

#### Updating rule 3: Position-dependent (PD) Bayesian S0

This updating rule is the same as rule 2, but learning a separate distribution for each of the eight possible target positions. All eight distributions start as the same starting distribution, a Beta distribution with the two parameters having the same value *β*_0_. However, after each trial, only the distribution associated with the target position on that trial was updated according to Eq 2. The forgetting mechanism (Eq 3) was applied to every position after each trial. This updating rule contributes two parameters, *α* and *β*_0_, to the model.

#### Updating rule 4: Position-gradient (PG) Bayesian S0

Similar to rule 2 and rule 3, this updating rule is in a sense intermediate between the two. Instead of either updating completely independently of position (like rule 2) or updating completely specific to a single position (like rule 3), updating was applied to every position on each trial, but the magnitude of the update decreased with the distance to the target position on that trial. The “partial” updates were implemented by using a weighted average between the Bernoulli likelihood and an equally distributed likelihood (note that applying Bayes rule with an equally distributed likelihood leaves the prior distribution unchanged and is therefore equivalent to not updating at all) for *P*(*X*_*t*_|*H*_*t*_) in Eq 2, where the weight given to the Bernoulli likelihood decreases exponentially with distance to the target position:

$$P(X_{t}|H_{t})=\omega^{d}p+(1-\omega^{d})/2,(\omega <1),$$ (4)

where d is the distance from the position being updated to the target position, ranging from d = 0 (the target position itself), through d = 1 (for each of the two neighbouring positions) to d = 4 (for the position on the opposite side of the circle).

The forgetting mechanism (Eq 3) was applied (equally) to every position after each trial. This updating rule contributes three parameters, α, ω and β_0_, to the model.

#### Updating rule 5: Position-spreading (PS) Bayesian S0

This updating rule was similar to rule 3 in that the originally completely separate probability distributions were learned for each position, but differed in that there was some degree of spreading of the learned distributions to other positions between trials. The spreading was applied immediately after determining the starting point biases on each trial, before updating the probability distributions based on the stimulus on that trial, so that the updates from one trial back had not yet been subjected to the spreading. The spreading was done by replacing the probability distribution associated with each position with a weighted average of the probability distributions from every position,

$$P_{i}=\sum_{j}w(d_{ij})P_{j}/\sum_{j}w(d_{ij}),$$ (5)

where P_i_ is the probability distribution associated with a particular position in the search display, the sum was performed over all positions in the search display, and d_ij_ is the distance between position i and j. The weight w decreased with the distance between positions according to a Gaussian distribution with zero mean and standard deviation *σ*:

$$w(d_{ij})=\frac{1}{\sigma \sqrt{2\pi }}e^{-d_{ij}^{2}/2\sigma^{2}}$$ (6)

This updating rule contributes three parameters, *α*, *σ* and *β*_0_, to the model.

#### Updating rule 6: Binary rate

Unlike updating rules 2–5 which all update the starting point, this rule applies to updating the evidence accumulation rate. This rule assumes that the rate only depends on whether there was a change of the updating variable from one trial back and not on the earlier trial history. The rate was decreased by scaling with *κ* (<1), if the updating variable has changed:

$$r_{n}=\kappa^{\left(1-\delta_{{UV}_{n},{UV}_{n-1}}\right)}r$$ (7)

where $\delta_{{UV}_{n},{UV}_{n-1}}$ is the Kronecker delta function applied to the values of the updating variable on trials n and n-1. This factor level contributes one parameter, *κ*, to the model.

#### Updating rule 7: Step rate

Like rule 6, this updating rule also assumes that the rate decreases after a switch of the updating variable compared to after a repetition. However, unlike rule 6, this rule assumes that these rate changes last for more than a single trial, but with memory decay. Specifically, the rate was scaled with *κ* on each trial, where *κ* has a starting value of (*κ*_0_ = 1), and then increases by *Δ* after each UV repetition and decreases by *Δ* after each UV switch. Also, the same forgetting mechanism as in rule 3 was used, so that *κ* was partially reset to the starting value (of 1) after each trial, thereby giving trials from further back less influence:

$$\kappa_{n+1}={\alpha *(\kappa }_{n}+(2\delta_{{UV}_{n},{UV}_{n-1}}-1)*\Delta )+(1-\alpha ){*\kappa }_{0}={\alpha *(\kappa }_{n}+(2\delta_{{UV}_{n},{UV}_{n-1}}-1)*\Delta )+(1-\alpha )$$ (8)

where $\delta_{{UV}_{n},{UV}_{n-1}}$ is the Kronecker delta function applied to the values of the updating variable on trials n and n-1. This updating rule contributes two parameters, *α* and *Δ* to the model.

#### Updating rule 8: Weighted rate

Like rule 7, this rule assumed that the rate was influenced by the entire history of the updating variable, with memory decay giving older trials less influence. However, this rule differed in that it used a separate scaling factor *κ*^(i)^ for each different level of the UV, and the *κ*^(i)^ always summed to a constant value, as if there was a shared ‘weight’ resource. After a trial on which a given value of the UV had occurred, some weight was moved to the scaling factor associated with that value of the UV and the same amount of weight was removed from all of the other scaling factors combined, removing an equal amount from each one, such that the total change summed to zero. In addition, the same forgetting mechanism used for rule 6 was applied. This updating rule was inspired by the dimension-weighting account [5,6]. Specifically, each scaling factor started as *κ*^(i)^ = 1, and after each trial they were updated as:

$$\kappa_{n+1}^{\left(i\right)}=\alpha (\kappa_{n}^{\left(i\right)}+\delta_{i,j}\Delta -{(1-\delta }_{i,j})(\frac{\Delta }{N-1}))+(1-\alpha ),$$ (9)

where *δ*_*i*,*j*_ is the Kronecker delta function, j is the index for the scaling factor associated with the value of the UV that occured on that trial and N is the total number of different possible values for that UV (two for the RCF and color, eight for position). This updating rule contributes two parameters, *α* and *Δ* to the model.

#### Updating rule 9: Weighted rate with distractor inhibition

This updating rule is similar to rule 8 but specific to position related updating. In addition to shifting weight to the target position, weight is shifted away from each of the three distractor positions. Like in rule 8 this is done in such a way that the sum of all changes is zero:

$$\kappa_{n+1}^{\left(i\right)}=\alpha (\kappa_{n}^{\left(i\right)}+\delta_{i,j}\Delta_{t}-{(1-\delta }_{i,j})(\frac{\Delta_{t}}{N-1})-\sum_{k\epsilon D_{n}}(\delta_{i,k}\frac{\Delta_{d}}{3}-(1-\delta_{i,k})\frac{\Delta_{d}}{5})+(1-\alpha ),$$ (10)

where *δ*_*i*,*j*_ is the Kronecker delta function, j is the index for the scaling factor associated with the value of the UV that occured on that trial, N is the total number of different possible values for the UV, and k is summed over the three distractor positions on trial n. This updating rule contributes three parameters, *α* and *Δ*_*t*_ and *Δ*_*d*_ to the model.

#### Updating rule 10: Weighted rate with matched distractor inhibition

This updating rule is similar to rule 9 but the increase in weight to the target position was exactly matched with the decrease in weight for the distractor positions and the weight of neutral (empty) positions was not changed:h matched distractor inhibition.

$$\kappa_{n+1}^{\left(i\right)}=\alpha (\kappa_{n}^{\left(i\right)}+\delta_{i,j}\Delta -\sum_{k\epsilon D_{n}}(\delta_{i,k}\frac{\Delta }{3}))+(1-\alpha )$$ (11)

where *δ*_*i*,*j*_ is the Kronecker delta function, j is the index for the scaling factor associated with the value of the UV that occured on that trial, and k is summed over the three distractor positions on trial n. This updating rule contributes two parameters, *α* and *Δ* to the model.

#### Updating rule 11: Binary NDT

This updating rule is similar to rule 6 in that it only takes into account whether there was a change of the UV from a single trial back, and ignores the older history, but instead of updating the evidence accumulation rate it is the non-decision time that is updated. The non-decision time is reduced by a constant amount *Δ* if the UV was repeated compared to if it changed from one trial back. This updating rule contributes one parameter, *Δ* to the model.

#### Updating rule 12: Weighted NDT

This updating rule is similar to rule 8, except that it updates the non-decision time instead of the rate. Also, instead of updating a scaling factor, the non-decision time is updated directly. In particular the non decision-time on trial n for level i of the updating variable was:

$$\tau_{n}^{\left(i\right)}=\tau_{0}+\delta \tau_{n}^{\left(i\right)},$$ (12)

where *τ*_0_ was a constant baseline component of the non-decision time and $\delta \tau_{n}^{\left(i\right)}$ started as $\delta \tau_{1}^{\left(i\right)}=0$ on trial 1 and was then updated after each trial by subtracting a constant amount from the $\delta \tau_{n}^{\left(i\right)}$ associated with the level of the UV which occured on that trial (thereby making the non-decision time associated with that level of the UV shorter) and adding the same amount distributed over the other levels of the UV. In addition the same forgetting mechanism used in rule 8 was applied, partially resetting $\delta \tau_{n}^{\left(i\right)}$ to the starting value of zero after each trial. These updates are described by the following equation:

$$\delta \tau_{n+1}^{\left(i\right)}=\alpha (\delta \tau_{n}^{\left(i\right)}-\delta_{i,j}\Delta {+(1-\delta }_{i,j})(\frac{\Delta }{N-1})),$$ (13)

where *δ*_*i*,*j*_ is the Kronecker delta function, j is the index for the scaling factor associated with the value of the UV that occured on that trial and N is the total number of different possible values for the UV (two for the RCF and color, eight for position). This updating rule contributes two parameters, *α* and *Δ* to the model.

#### Updating rule 13: Weighted NDT with distractor inhibition

This updating rule is similar to rule 10 but specific to position related updating (like rule 9). In addition to shifting weight to the target position, weight is shifted away from each of the three distractor positions. Like in rule 12 this is done in such a way that the sum of all changes is zero:

$$\delta \tau_{n+1}^{\left(i\right)}=\alpha (\delta \tau_{n}^{\left(i\right)}-\delta_{i,j}\Delta_{t}{+(1-\delta }_{i,j})(\frac{\Delta_{t}}{N-1})+\sum_{k\epsilon D_{n}}(\delta_{i,k}\frac{\Delta_{d}}{3}-(1-\delta_{i,k})\frac{\Delta_{d}}{5})),$$ (14)

where *δ*_*i*,*j*_ is the Kronecker delta function, j is the index for the scaling factor associated with the value of the UV that occured on that trial, N is the total number of different possible values for the UV, and k is summed over the three distractor positions on trial n. This updating rule contributes three parameters, *α* and *Δ*_*t*_ and *Δ*_*d*_ to the model.

#### Updating rule 14: Weighted NDT with matched distractor inhibition

This updating rule is similar to rule 13 except the decrease in NDT to the target position was exactly matched with the sum of increase in NDT over the three distractor positions and the neutral (empty) positions were not changed:

$$\delta \tau_{n+1}^{\left(i\right)}=\alpha (\delta \tau_{n}^{\left(i\right)}-\delta_{i,j}\Delta +\sum_{k\epsilon D_{n}}(\delta_{i,k}\frac{\Delta }{3})),$$ (15)

where *δ*_*i*,*j*_ is the Kronecker delta function, j is the index for the scaling factor associated with the value of the UV that occured on that trial, and k is summed over the three distractor positions on trial n. This updating rule contributes two parameters, *α* and *Δ* to the model.

#### Updating rule 15: Position dependent (PD) step NDT

This rule is similar to rule 3, in that updating only affects the position where the target occured, but applied to the non-decision time instead of the starting point, and with updating and memory dynamics similar to rule 7, in that repetitions/switches of the UV from trial to trial result in decreases/increases of *δτ* which are partially remembered across trials. A separate *δτ* is updated for each position, according to:

$$\delta \tau_{n+1}=\alpha (\delta \tau_{n}+(2\delta_{{UV}_{n},{UV}_{n-1}}-1)*\Delta ),$$ (16)

where $\delta_{{UV}_{n},{UV}_{n-1}}$ is the Kronecker delta function applied to the UV on trial n-1 and the UV on trial n (so $\left(2\delta_{{UV}_{n},{UV}_{n-1}}-1\right)$ equals 1 for repeated UV and -1 for UV switch). This updating rule contributes two parameters, *α* and *Δ* to the model.

#### Updating rule 16: Position-gradient (PG) step NDT

This rule is similar to rule 4, but updating the non-decision time instead of the starting point. A separate *δτ* is updated for each position, such that each update most strongly affects the target position but also affects other positions with the size of the update decaying with distance:

$$\delta \tau_{n+1}=\alpha (\delta \tau_{n}+(2\delta_{{UV}_{n},{UV}_{n-1}}-1)*\omega^{d}\Delta ),(\omega <1),$$ (17)

where d is the distance from the position being updated to the target position, defined the same way as for rule 6, and $\delta_{{UV}_{n},{UV}_{n-1}}$ is the Kronecker delta function applied to the UV on trial n-1 and the UV on trial n. This updating rule contributes three parameters, *α*, ω, *and Δ* to the model.

#### Updating rule 17: Position-dependent (PD) weighted rate

This rule is similar to rule 8, except that, similar to rule 3, updating is done separately for each position. Like rule 8 it used a separate scaling factor for each different level of the UV, but unlike rule 8, these scaling factors were also different for each position: $\kappa_{n+1}^{\left(i,p\right)}$ where i represents the level of the UV and p the position. For each position, the scaling factors summed to a constant value (summing over the different levels of the UV). After each trial, the scaling factor associated with the target position on that trial was updated according to Eq 9 while only the forgetting rule was applied to the scaling factors associated with different positions:

$$\kappa_{n+1}^{\left(i,p\right)}=\alpha \kappa_{n}^{\left(i,p\right)}+(1-\alpha )\;for\;p\neq p_{n}^{t},$$ (18)

where $p_{n}^{t}$ is the target position on trial n. This updating rule contributed two parameters, *α* and *Δ* to the model.

#### Updating rule 18: Position-gradient (PG) weighted rate

This rule is similar to rule 8, except that, like for rule 17, scaling factors were different for each position: $\kappa_{n+1}^{\left(i,p\right)}$ and, similar to rule 4, the magnitude of the update decreased with the distance to the target position. After each trial, the scaling factors were updated by shifting weight to the level of the UV which occured on that trial, with the size of the update decreasing with distance:

$$\kappa_{n+1}^{\left(i,p\right)}=\alpha (\kappa_{n}^{\left(i,p\right)}+{\omega^{d}(2\delta }_{i,j}-1)\Delta )+(1-\alpha ),$$ (19)

where *δ*_*i*,*j*_ is the Kronecker delta function, j is the index for the scaling factor associated with the value of the UV that occured on that trial, and d is the distance from the position being updated to the target position, defined the same way as for rule 4.

### Model comparison

Given that our model had four factors with 2, 12, 8, and 10 levels (evidence accumulation model, RCF-based updating, color-based updating, and position-based updating), there were a total of 1920 possible combinations of the factor levels. Each combination considered had to be fit to each of the two sessions for 14 participants. If each possible combination of factor levels had been considered, this would have resulted in a total of 26880 model fits. Since this would have been computationally too demanding, we used the following procedure to find a model which cannot be improved by changing any single factor:

1. For each of the three updating rule factors, the model was fitted to the data from both sessions for all participants, for each level of that factor, with the other two updating rule factors fixed to the “no updating” level. This was done for both levels of the evidence accumulation (EA) model factor (LATER model and DDM).
2. For each of the three updating rule factors, the mean AIC was compared between the different factor levels, finding the best factor level for that factor in terms of the lowest AIC, across both levels of the EA model factor.
3. Steps 1 and 2 were repeated but now fixing factors to the “best” levels discovered in (the previous repetition of) step 2 instead of to the “no updating” level. This repetition continued until the best factor level for each factor was the same as in the previous repetition. This happened on the third repetition, that is, there was no change between the second and third repetitions, and only the position-based updating factor changed between the first and second repetition.

## Supporting information

S1 Appendix Model comparison based on individual participants. (PDF)

S2 Appendix Parameters of the best model. (PDF)

S3 Appendix Predictions of non-winning models. (PDF)

S4 Appendix Three stage model. (PDF)

S5 Appendix Reanalysis of EEG data from Gokce et al. (2015). (PDF)

S6 Appendix Cross-validation—out-of-fold model predictions. (PDF)
