# Supplementary material for: Inter-trial effects in priming of pop-out: Comparison of computational updating models
Source: PLoS Comput Biol. 2021 Sep 3;17(9):e1009332. doi: 10.1371/journal.pcbi.1009332 (PMC8445473; doi:10.1371/journal.pcbi.1009332)
Supplement: S1 Appendix — (PDF) [file pcbi.1009332.s001.pdf]

S1 Appendix: Model comparison based on individual participants

Here we consider a different approach of comparing models: pick the best model for each participant and session, and count how many times each level of each factor occurs in the resulting list. Table A below shows the results of this analysis. The table has four sections: one for the evidence accumulation model, LATER model vs. DDM, and one for each updating variable: color-based updating, position-based updating and RCF-based updating.

For the two candidates of the evidence accumulation model, we found that 82% of the best fitting models favored the LATER model than the DDM model. This is consistent with the analysis based on average AIC-values. For the color-based feature priming of pop-out, the majority of best-fitting models (89%) used the (position independent) weighted rate updating rule, while only 4% used an updating rule for the non-decision time. The proportion was reduced for the positional priming of pop-out, about 71% of the best-fitting models using the weighted rate with distractor inhibition rule while 29% using rules based on updating the non-decision time. Consistent with this there was also less difference between the winning rule and the best rule based on non-decision time in the analysis based on average AIC-values. This suggests that, while position-based inter-trial effects may mostly be based on trial to trial changes in the evidence accumulation rate, the non-decision time may also play an important role. Finally, 61% of the best-fitting models for RCF-based updating applied the position-gradient (PG) Bayesian starting point updating rule, with 11% using updating of the non-decision time and 28% using the position-spreading (PS) Bayesian starting point updating rule. Interestingly, no updating rule based on the rate was used in any of the best-fitting models.

Overall these results are consistent with the analysis performed based on average AIC, in that, for each factor, the model with the lowest average AIC also appeared in the majority of best-fitting models.

Table A: *Model comparison across individual participants and sessions*

Model	
RT distribution model: DDM	5
RT distribution model: LATER	23
Color: No update	0
Color: PI Binary rate	1
Color: PI Step rate	1
Color: PI Weighted rate	25
Color: PI Binary NDT	0
Color: PI Weighted NDT	1
Color: PD Weighted rate	0
Color: PG Weighted rate	0
Position: No update	0
Position: Binary rate	0
Position: Step rate	0
Position: Weighted rate	0
Position: Weighted rate with dist. inhib.	20
Position: Weighted rate with matched dist. inhib.	0
Position: Binary NDT	2
Position: Weighted NDT	3
Position: Weighted NDT with dist. inhib	3
Position: Weighted NDT with matched dist. inhib	0
Response: No update	2
Response: PI Bayesian S0	0
Response: PD Bayesian S0	0
Response: PG Bayesian S0	17
Response: PS Bayesian S0	6
Response: PI binary rate	0
Response: PI step rate	0
Response: PI weighted rate	0
Response: PI binary NDT	0
Response: PI weighted NDT	1
Response: PD step NDT	1
Response: PG step NDT	1
