# Supplementary material for: Inter-trial effects in priming of pop-out: Comparison of computational updating models
Source: PLoS Comput Biol. 2021 Sep 3;17(9):e1009332. doi: 10.1371/journal.pcbi.1009332 (PMC8445473; doi:10.1371/journal.pcbi.1009332)
Supplement: S2 Appendix — (PDF) [file pcbi.1009332.s002.pdf]

S2 Appendix: Parameters of the best model

The model comparison revealed that the best model used the Position-gradient (PG) Bayesian S0 rule (updating rule 4 in the "Models and updating rules" subsection of the Methods section) for updating based on the RCF, the position-independent weighted rate rule (updating rule 7) for updating based on target color, and the weighted rate with distractor inhibition rule (updating rule 8) for updating based on position. Here, we examine the parameters that provided the best model fits for each of these rules. Table B shows the mean and standard deviation (SD), across participants and the two experimental sessions, of each parameter.

Table B: *Mean and standard deviation of the updating rule parameters of the best model*

Updating variable	Response-critical feature			Color		Position		
Parameter	α	β_0	ω	α	Δ	α	Δ_t	Δ_d
Mean	0.78	4.23	0.52	0.53	0.15	0.63	0.13	0.11
SD	0.28	6.83	0.31	0.22	0.23	0.23	0.19	0.19

The αparameters capture the memory decay and can range from 0 to 1, with smaller values indicating faster decay. The β_0 parameter defines the shape of the beta-distributed hyperprior of the Bayesian updating, with smaller values being indicative of faster updating. The ω parameter defines how localized the updating in the position-gradient rule was; it can range from 0 to 1, with smaller values indicating more localized updating. The Δ, Δ_t, and Δ_d parameters define the size of the update of the scaling factors (that determine the evidence accumulation rate) for the target color, target position, and distractor positions, respectively. See the "Models and updating rules" subsection of the Methods section for more details on the updating rules and the precise meaning of the parameters.
