# Supplementary material for: Inter-trial effects in priming of pop-out: Comparison of computational updating models
Source: PLoS Comput Biol. 2021 Sep 3;17(9):e1009332. doi: 10.1371/journal.pcbi.1009332 (PMC8445473; doi:10.1371/journal.pcbi.1009332)
Supplement: S3 Appendix — (PDF) [file pcbi.1009332.s003.pdf]

S3 Appendix: Predictions of non-winning models

Here we consider the predicted temporal profiles of the inter-trial effects based on models other than the winning model. In particular we consider the predictions when one of the factors in the model comparison is changed while using the factor levels of the best model for the other three. The winning model factor levels were LATER model with response based updating using the "PG Bayesian S0" rule, color based updating based on the "weighted rate" rule, and position based updating based on the "weighted rate with distractor inhibition" rule. Each of the models below deviate from this combination in terms of one of the factors, as explained in the text below.

Color based models

Each of the models presented in this section deviates from the winning model in terms of the color based updating rule.

No update

Fig A shows the predictions of the "No update" rule which predicts no intertrial effects.

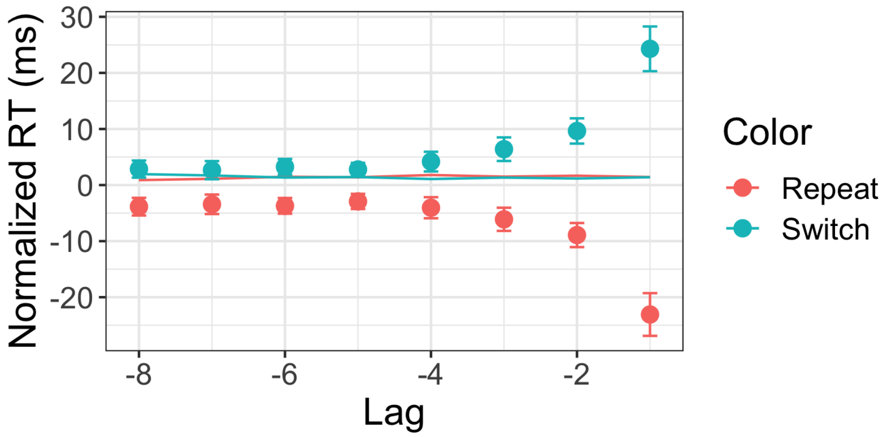

Fig A: Temporal profile of the color-based inter-trial effects with model predictions based on the "No update" rule for color based updating.

PI binary rate

Fig B shows the predictions of the "PI binary rate" rule which predicts inter-trial effects well from a single trial back, but predicts no inter-trial effects from further back.

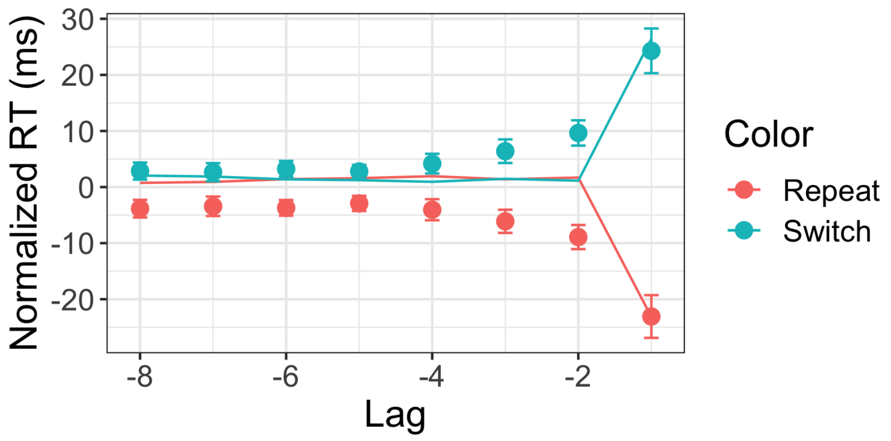

Fig B**:** Temporal profile of the color-based inter-trial effects with model predictions based on the "PI binary rate" rule for color based updating**.**

PI step rate

Fig C shows the predictions of the "PI step rate" rule which has a memory, but because it is the wrong kind of memory the predictions, with optimal parameter values, are essentially the same as with the "PI binary rate" rule.

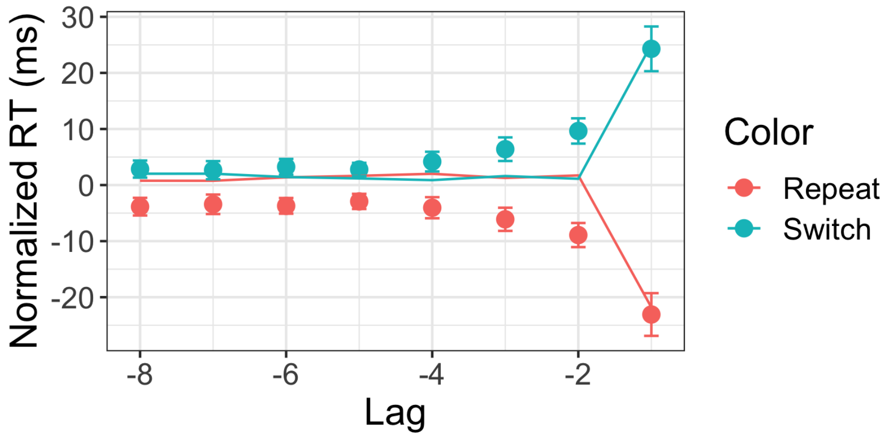

Fig C**:** Temporal profile of the color-based inter-trial effects with model predictions based on the "PI step rate" rule for color based updating**.**

PI binary NDT

Fig D shows the predictions of the "PI binary NDT" rule which predicts no inter-trial effects from more than one trial back, and additionally underestimates the inter-trial effects from a single trial back.

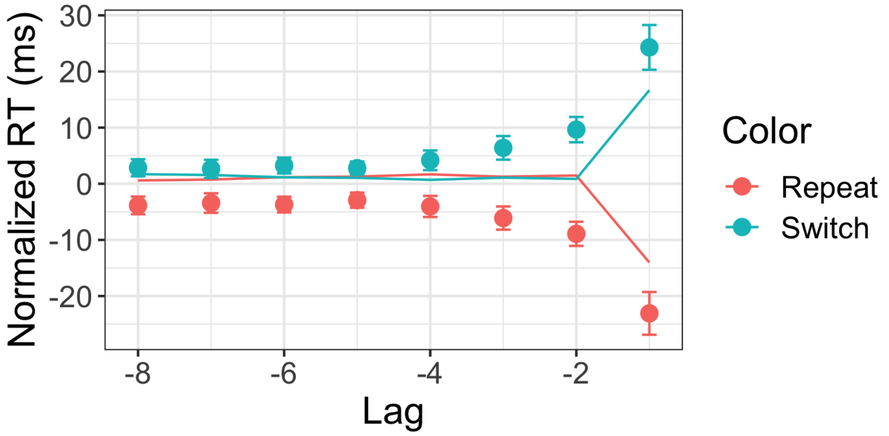

Fig D**:** Temporal profile of the color-based inter-trial effects with model predictions based on the "PI binary NDT" rule for color based updating**.**

PI weighted NDT

Fig E shows the predictions of the "PI weighted NDT" rule, which underestimates the inter-trial effects, in particular from a single trial back.

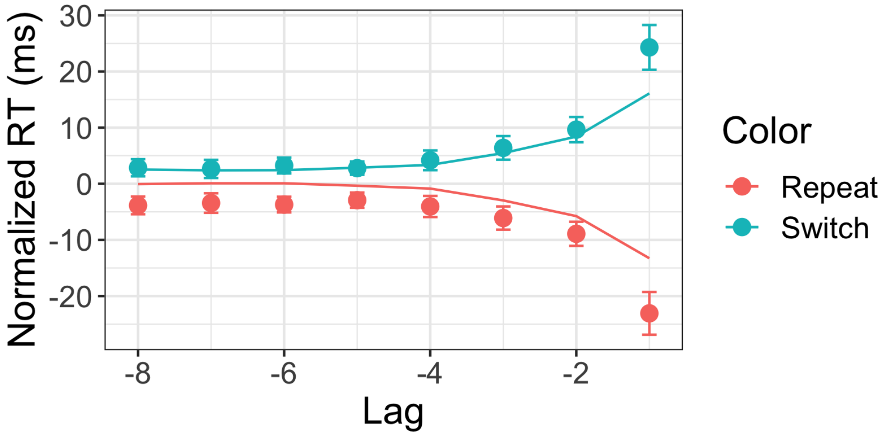

Fig E**:** Temporal profile of the color-based inter-trial effects with model predictions based on the "PI weighted NDT" rule for color based updating**.**

For the last two color-based update rules we show the inter-trial effects separately for the different positional inter-trial transition conditions since the predictions of these updating rules depend on whether the position is repeated.

PD weighted rate

Fig F shows the predictions of the PD weighted rate rule, which predicts the inter-trial effects well when the target position is repeated, but incorrectly predicts no inter-trial effects when the position is not repeated.

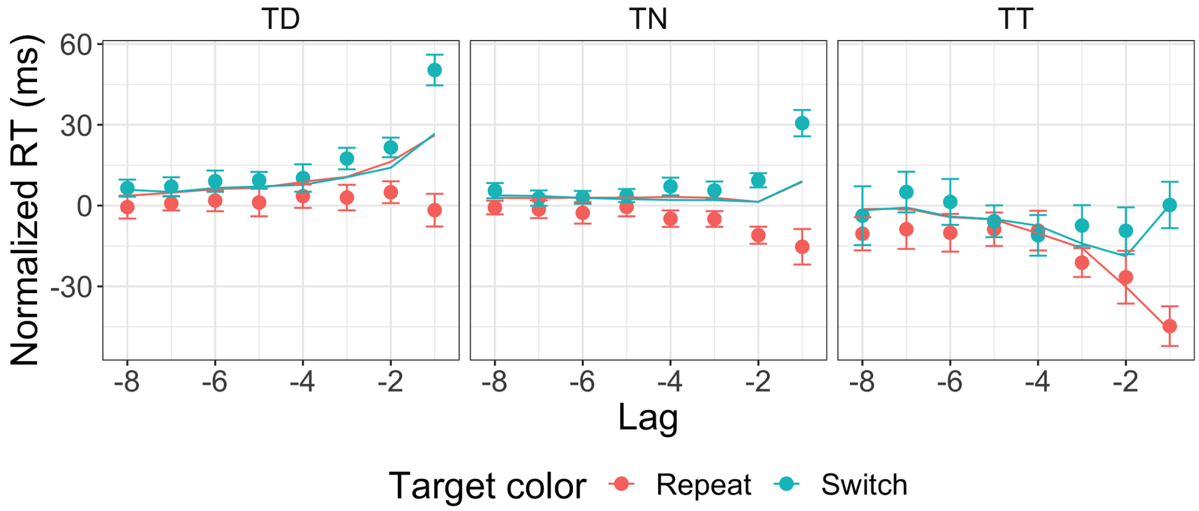

Fig F**:** Temporal profile of the color-based inter-trial effects with model predictions based on the "PD weighted rate" rule for color based updating**.**

PG weighted rate

Fig G shows the predictions of the PG weighted rate rule. This update rule predicts the inter-trial effects quite well, but fits the data no better than the winning rule while requiring an additional parameter.

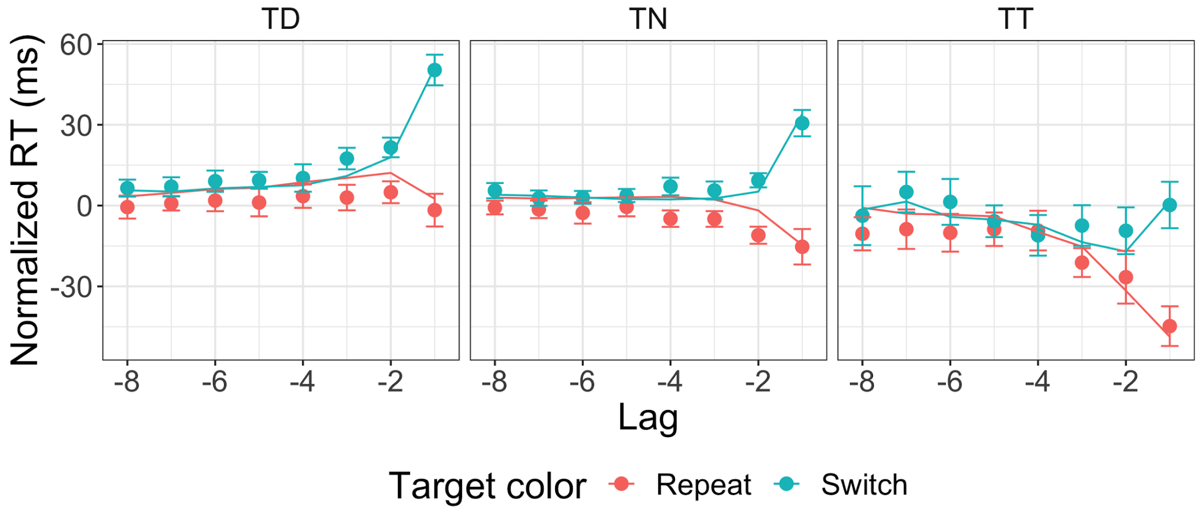

Fig G**:** Temporal profile of the color-based inter-trial effects with model predictions based on the "PG weighted rate" rule for color based updating**.**

Position based models

Each of the models presented in this section deviates from the winning model in terms of the position based updating rule.

No update

Fig H shows the predictions of the "No update" rule which predicts no intertrial effects.

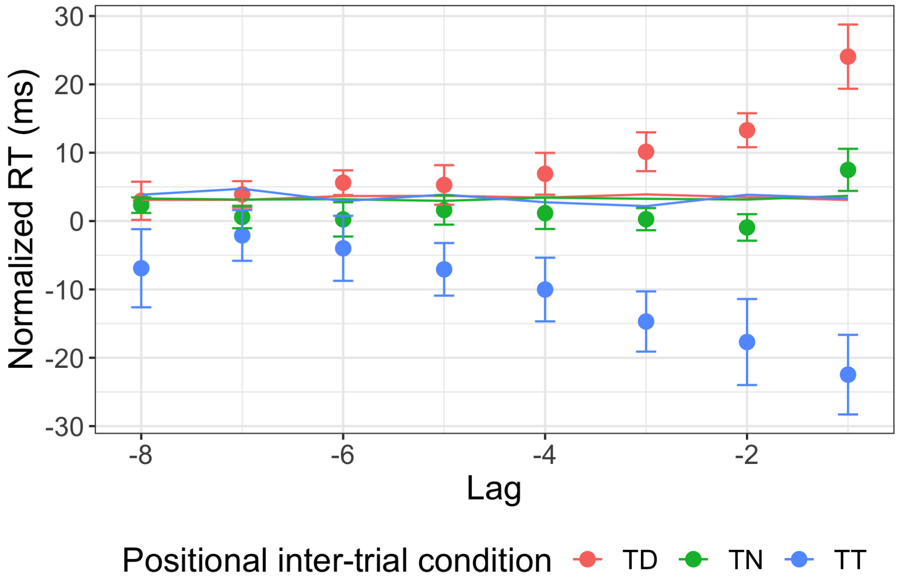

Fig H: Temporal profile of the position-based inter-trial effects with model predictions based on the “No update” rule for position based updating.

Binary rate

Fig I shows the predictions of the “Binary rate” rule which predicts the target position repetition benefit from a single trial back well, but underestimates inter-trial effects from more than one trial back and does not capture the difference between the target in previous distractor location and target in previous neutral location conditions. The prediction of a small inter-trial effect from two trials back is not a result of an actual memory from two trials back in the model, but an artifact of data processing resulting from the unequal position transition probabilities. Because the target had a larger probability of remaining in the same position compared to moving to any other particular position trial sequences with the same target position on trial n-2 and trial n more often also had the same target position on trial n-1 as on trial n compared to in sequences where the target positions on trials n-2 and trial n were different.

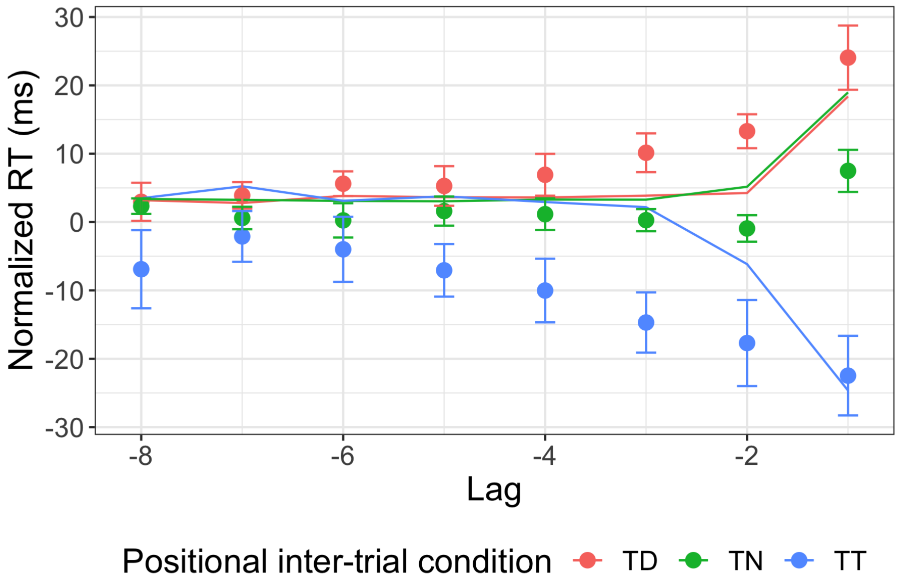

Fig I: Temporal profile of the position-based inter-trial effects with model predictions based on the “Binary rate” rule for position based updating.

Step rate

Fig J shows the predictions of the “Step rate” rule which, due to having the wrong type of memory, makes similar predictions to the “Binary rate” rule with no memory.

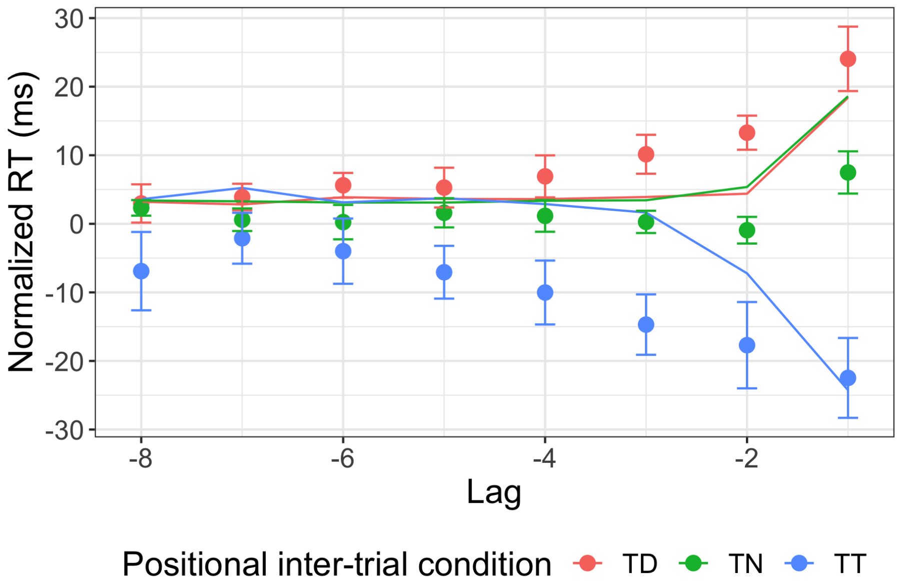

Fig J**:** Temporal profile of the position-based inter-trial effects with model predictions based on the "Step rate" rule for position based updating**.**

Weighted rate

Fig K shows the predictions of the "Weighted rate" rule which predicts the target position repetition benefits well, but does not capture the difference between the target in previous distractor location and target in previous neutral location conditions.

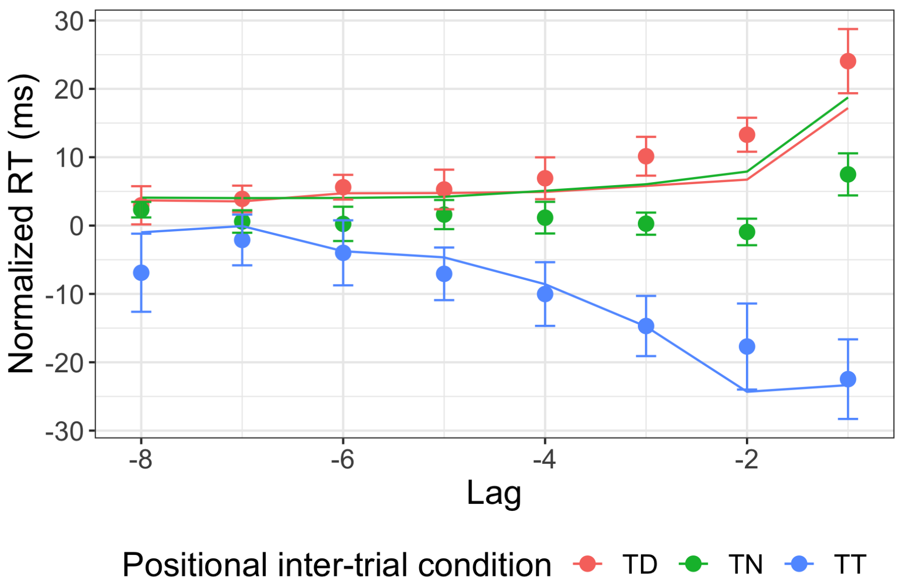

Fig K**:** Temporal profile of the position-based inter-trial effects with model predictions based on the “Weighted rate” rule for position based updating**.**

Matched weighted rate

Fig L shows the predictions of the “Matched weighted rate” rule, which captures inter-trial effects from a single trial back well, but underestimates older inter-trial effects, perhaps because the effect of shifting weight to and from neutral locations, which this rule did not do, gradually builds up over multiple trials.

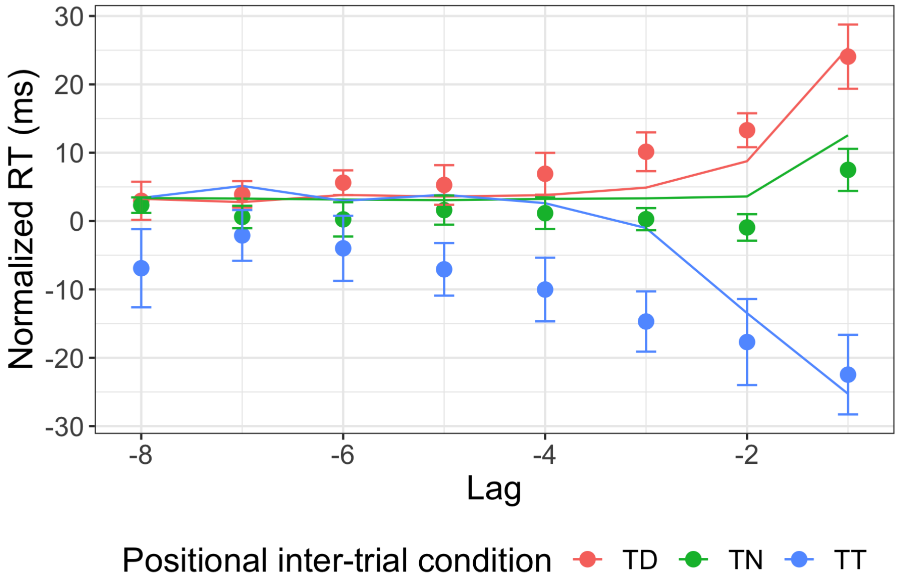

Fig L**:** Temporal profile of the position-based inter-trial effects with model predictions based on the “Matched weighted rate” rule for position based updating**.**

Binary NDT

Fig M shows the predictions of the “Binary NDT” rule, which underestimates inter-trial effects, in particular from more than one trial back (see the “Binary rate” rule for an explanation of why it “predicts” any inter-trial effects at all from more than one trial back), and does not capture the difference between the target in previous distractor location and target in previous neutral location conditions.

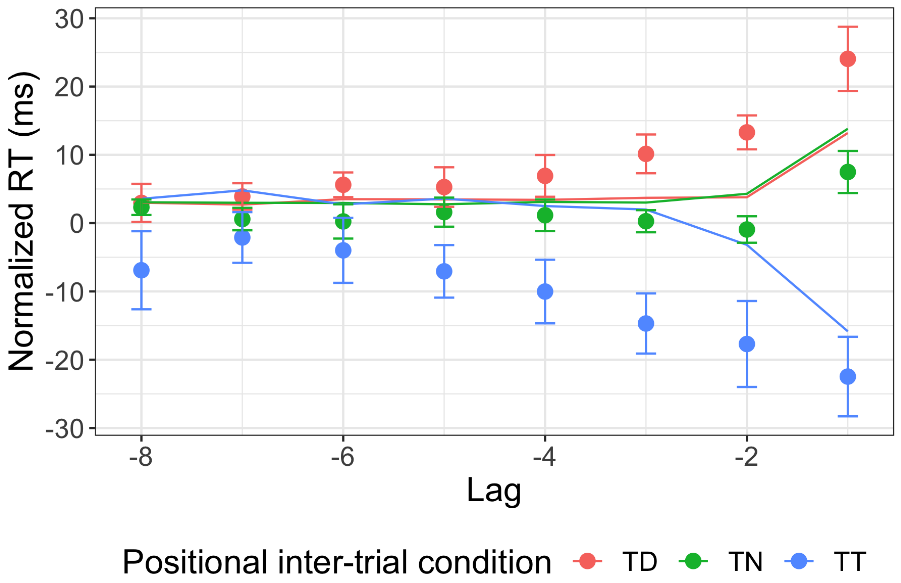

Fig M**:** Temporal profile of the position-based inter-trial effects with model predictions based on the "Binary NDT" rule for position based updating**.**

Weighted NDT

Fig N shows the predictions of the "Weighted NDT" rule which predicts the target position repetition benefits well, but does not capture the difference between the target in previous distractor location and target in previous neutral location conditions.

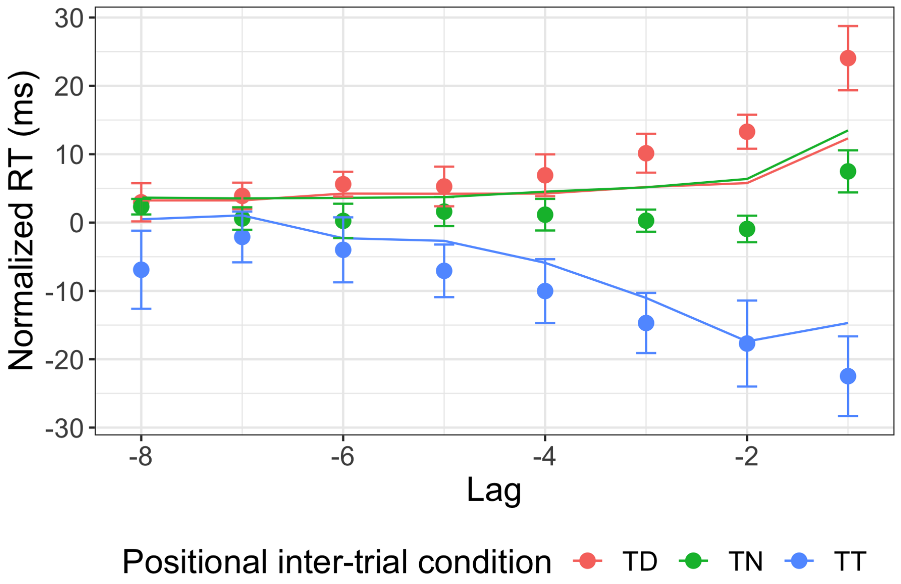

Fig N**:** Temporal profile of the position-based inter-trial effects with model predictions based on the "Weighted NDT" rule for position based updating**.**

Weighted NDT with DI

Fig O shows the predictions of the "Weighted NDT" rule which predicts the inter-trial effects quite well, although it slightly underestimates them compared to the winning rule.

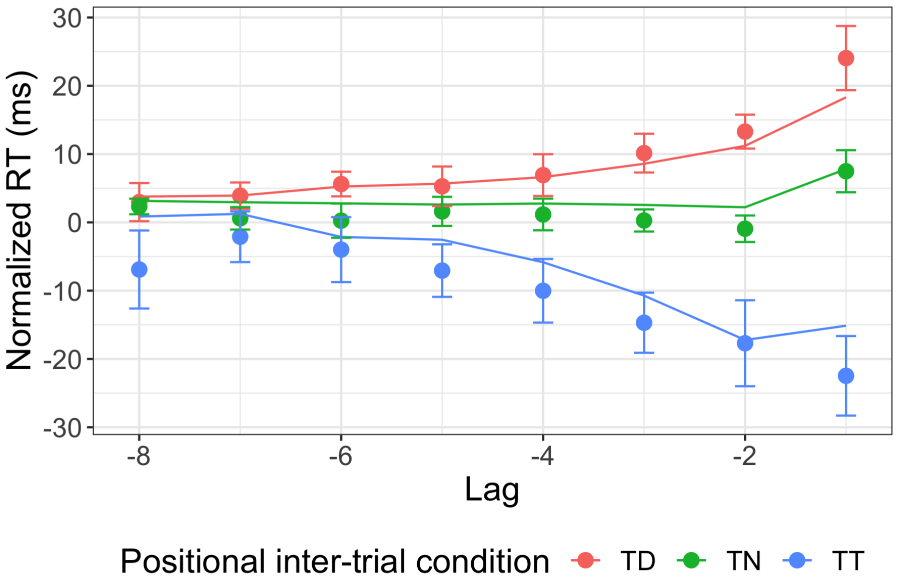

Fig O**:** Temporal profile of the position-based inter-trial effects with model predictions based on the "Weighted NDT with DI" rule for position based updating**.**

Matched weighted NDT

Fig P shows the predictions of the "Matched weighted NDT" rule which underestimates the inter-trial effects, in particular from more than one trial back (see "Matched weighted rate" for a discussion of a possible reason why).

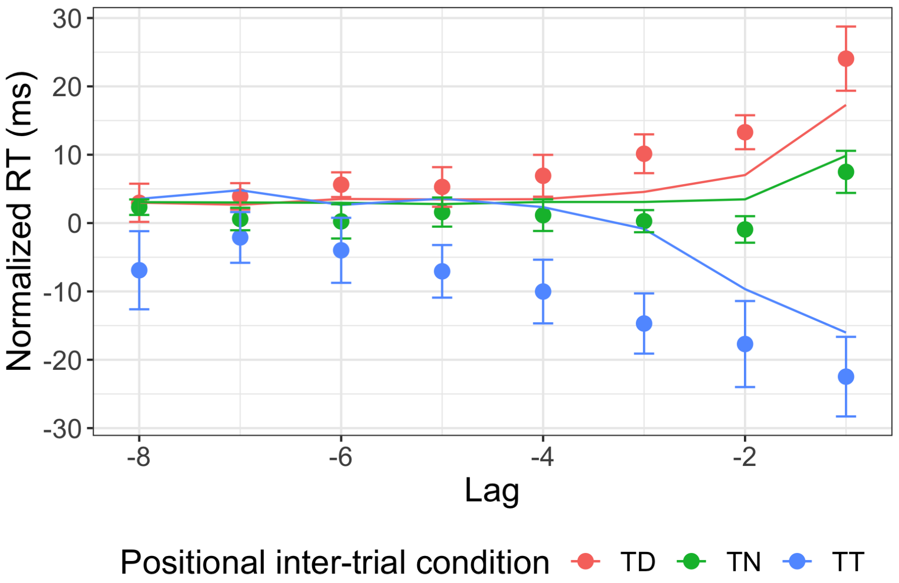

Fig P**:** Temporal profile of the position-based inter-trial effects with model predictions based on the “Matched weighted NDT” rule for position based updating**.**

Response based models

Each of the models presented in this section deviates from the winning model in terms of the response based updating rule.

No update

Fig Q shows the predictions of the “No update” rule which predicts no response-based intertrial effects, although position based intertrial effects are predicted since the “No update” rule for response-based updating was combined with the “Weighted rate with DI” rule for position-based updating.

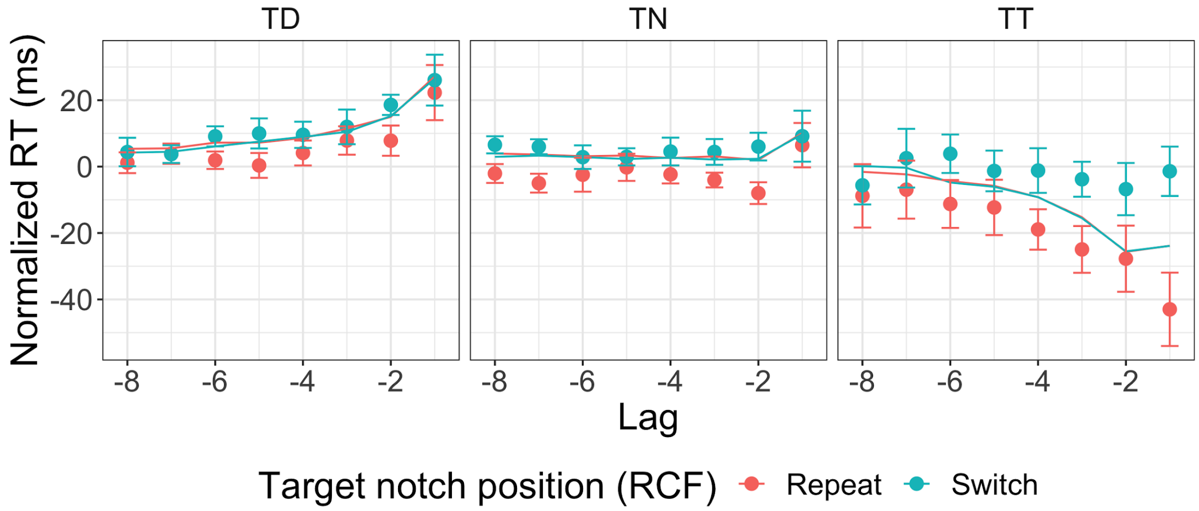

Fig Q**:** Temporal profile of the response-based inter-trial effects with model predictions based on the "No update" rule for response based updating**.**

PI Bayesian S0

Fig R shows the predictions of the "PI Bayesian S0" rule which underestimates inter-trial effects in the repeated target position condition and overestimates the inter-trial effects in the conditions in which the target position was not repeated.

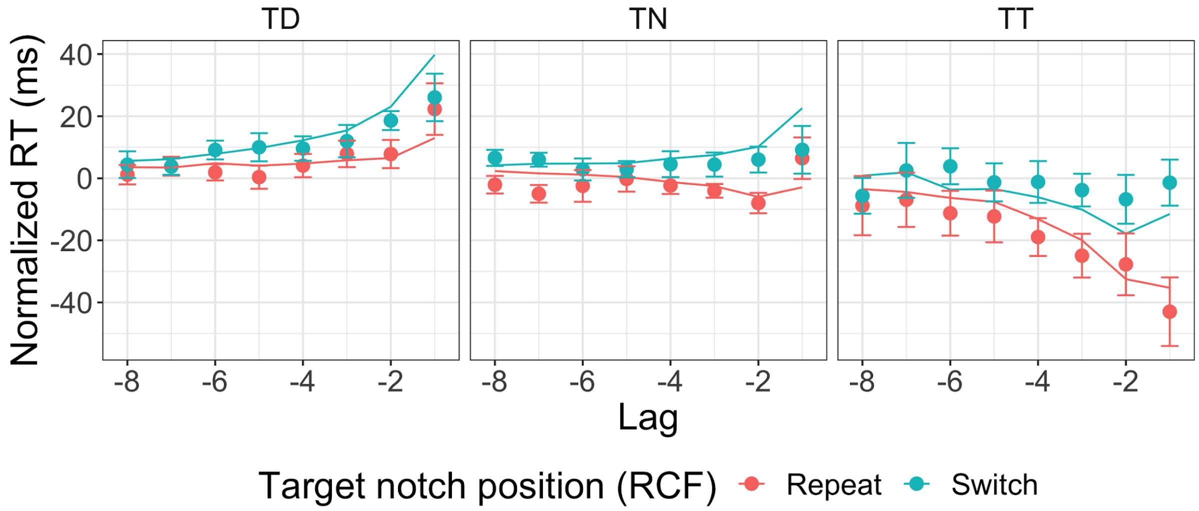

Fig R**:** Temporal profile of the response-based inter-trial effects with model predictions based on the "PI Bayesian S0" rule for response based updating**.**

PI binary rate

Fig S shows the predictions of the “PI binary rate” rule which incorrectly predicts no (response-based) inter-trial effects from more than a single trial back.

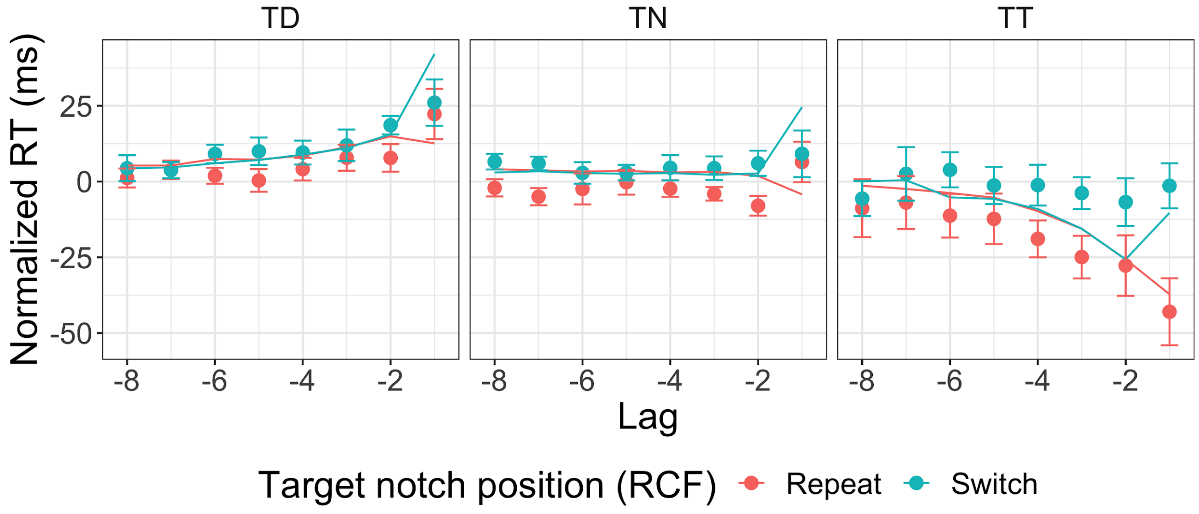

Fig S**:** Temporal profile of the response-based inter-trial effects with model predictions based on the “PI binary rate” rule for response based updating**.**

PI step rate

Fig T shows the predictions of the “PI step rate” rule which incorrectly predicts no (response-based) inter-trial effects from more than a single trial back, probably because this rule had the wrong type of memory.

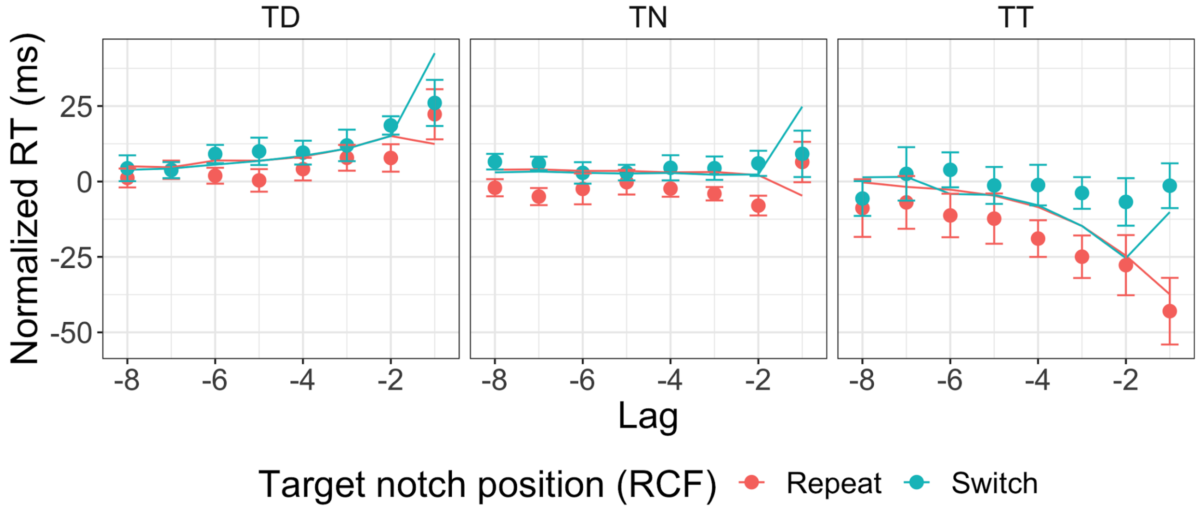

Fig T: Temporal profile of the response-based inter-trial effects with model predictions based on the "PI step rate" rule for response based updating.

PI weighted rate

Fig U shows the predictions of the "PI weighted rate" rule which underestimates inter-trial effects in the repeated target position condition and overestimates the inter-trial effects in the conditions in which the target position was not repeated.

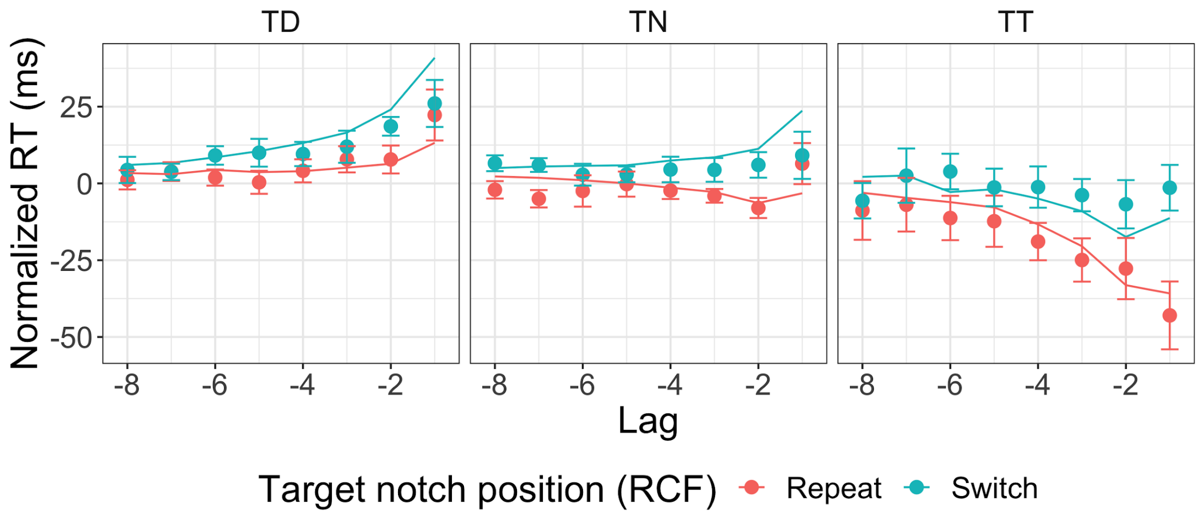

Fig U: Temporal profile of the response-based inter-trial effects with model predictions based on the "PI weighted rate" rule for response based updating.

PI binary NDT

Fig V shows the predictions of the "PI binary NDT" rule which incorrectly predicts no (response-based) inter-trial effects from more than a single trial back.

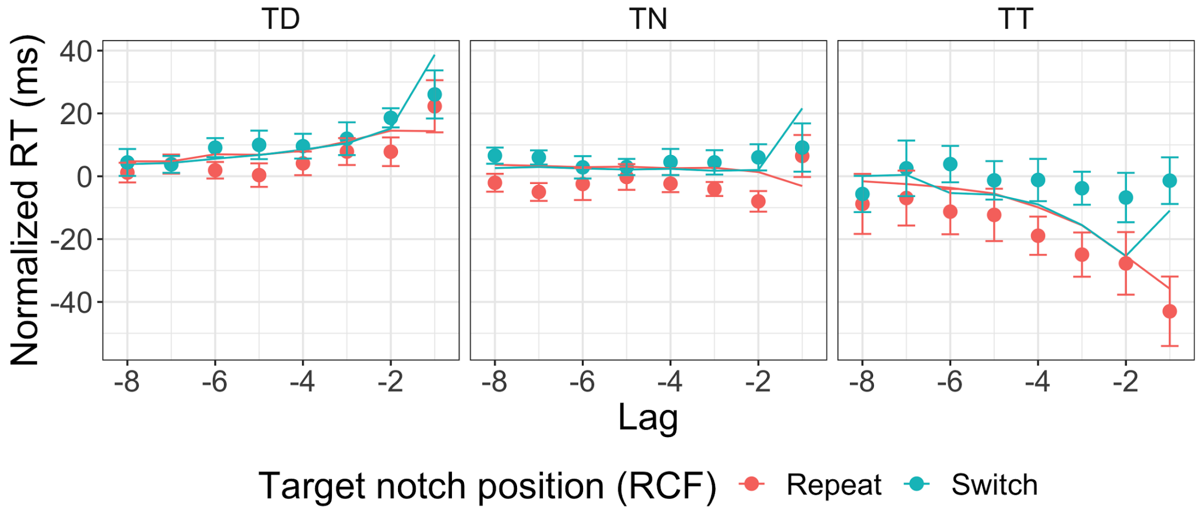

Fig V**:** Temporal profile of the response-based inter-trial effects with model predictions based on the "PI binary NDT" rule for response based updating**.**

PI weighted NDT

Fig W shows the predictions of the "PI weighted NDT" rule which underestimates inter-trial effects in the repeated target position condition and overestimates the inter-trial effects in the conditions in which the target position was not repeated.

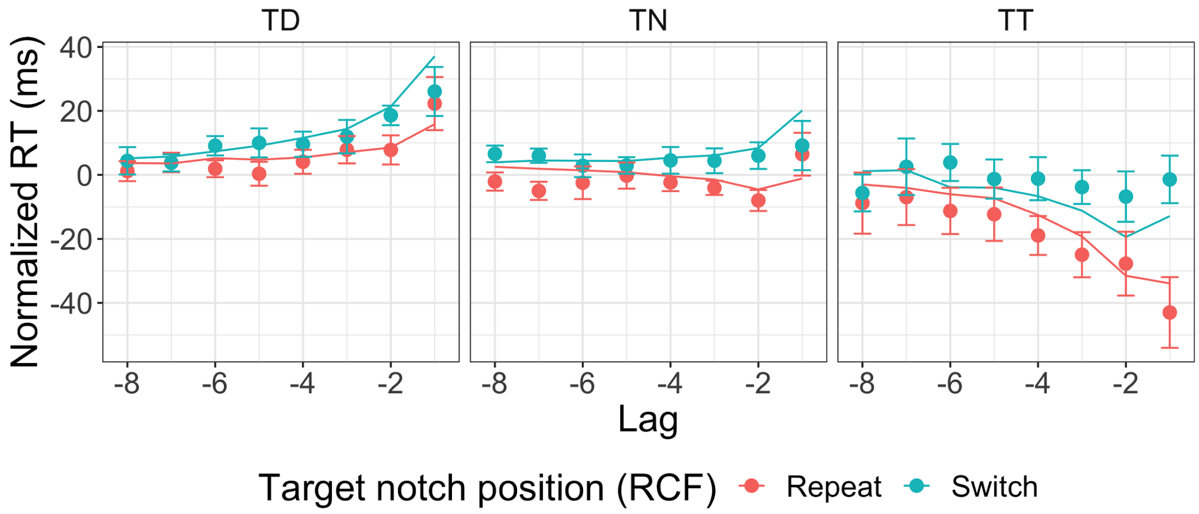

Fig W**:** Temporal profile of the response-based inter-trial effects with model predictions based on the "PI weighted NDT" rule for response based updating**.**

PI Bayesian S0

Fig X shows the predictions of the “PI Bayesian S0” rule which predicts the inter-trial effects in the repeated target position condition well but incorrectly predicts that there should be no (response based) inter-trial effects in the conditions in which the target position was not repeated.

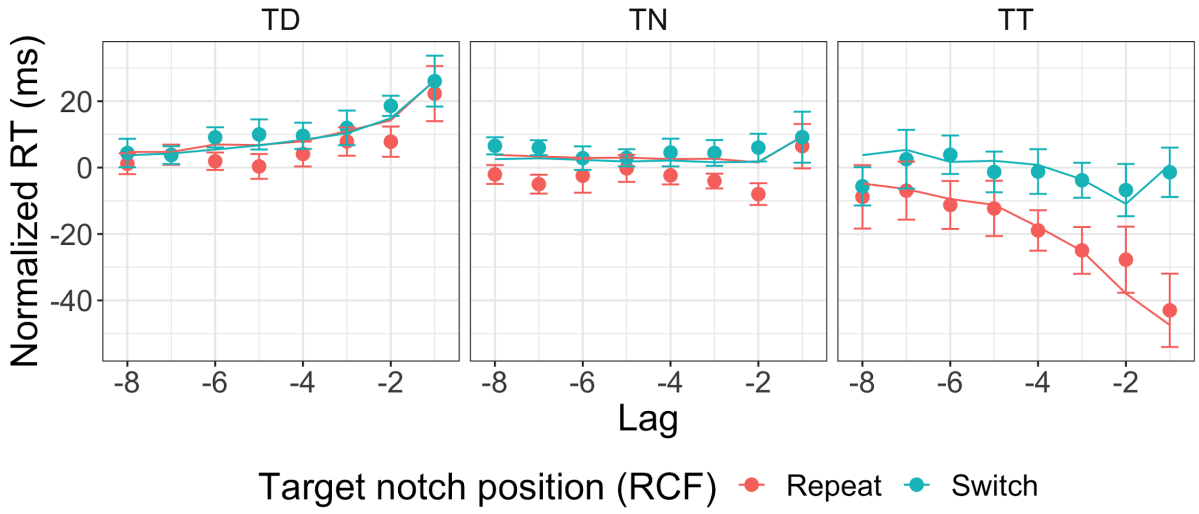

Fig X: Temporal profile of the response-based inter-trial effects with model predictions based on the “PD Bayesian S0” rule for response based updating**.**

PS Bayesian S0

Fig Y shows the predictions of the “PS Bayesian S0” rule which predicts the inter-trial effects from a single trial back well, but underestimates the inter.trial effects from more than one trial back in the repeated target position condition.

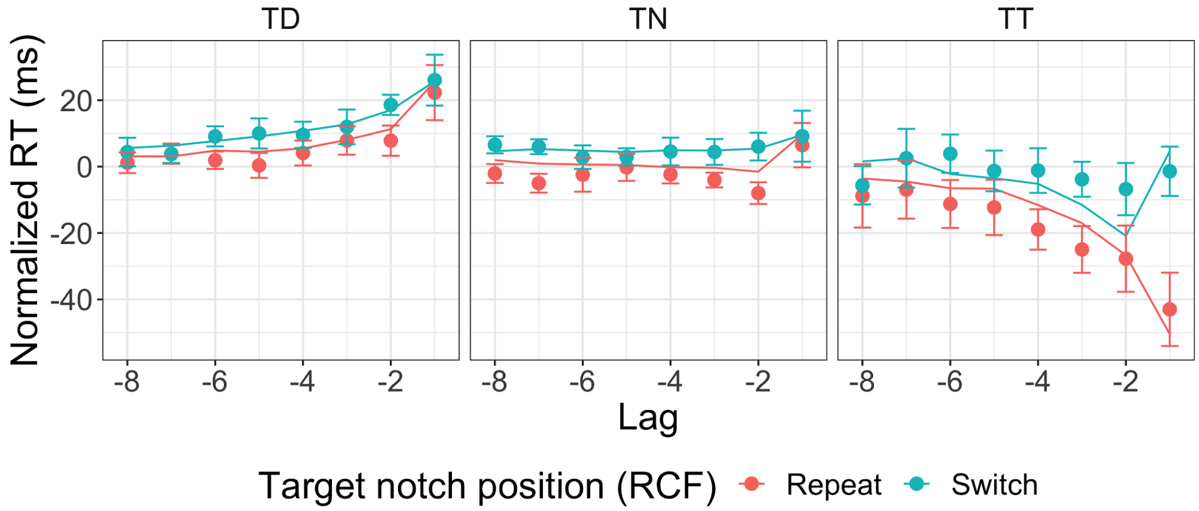

Fig Y: Temporal profile of the response-based inter-trial effects with model predictions based on the "PS Bayesian S0" rule for response based updating.

PD step NDT

Fig Z shows the predictions of the "PD step NDT" rule which incorrectly predicts no (response-based) inter-trial effects from more than a single trial back.

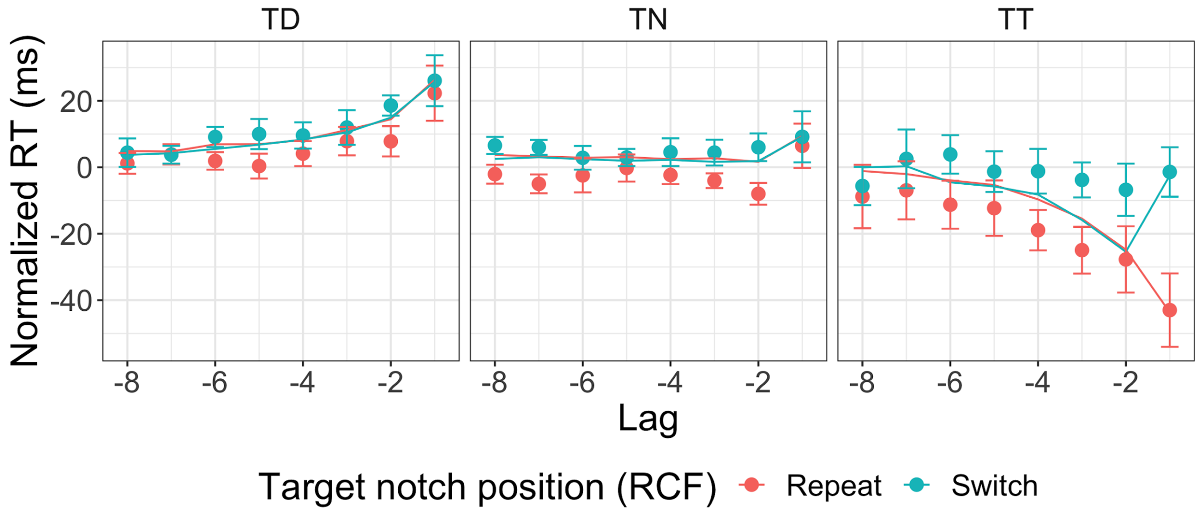

Fig Z: Temporal profile of the response-based inter-trial effects with model predictions based on the "PD Step NDT" rule for response based updating.

PG step NDT

Fig AA shows the predictions of the “PG step NDT” rule which incorrectly predicts no (response-based) inter-trial effects from more than a single trial back.

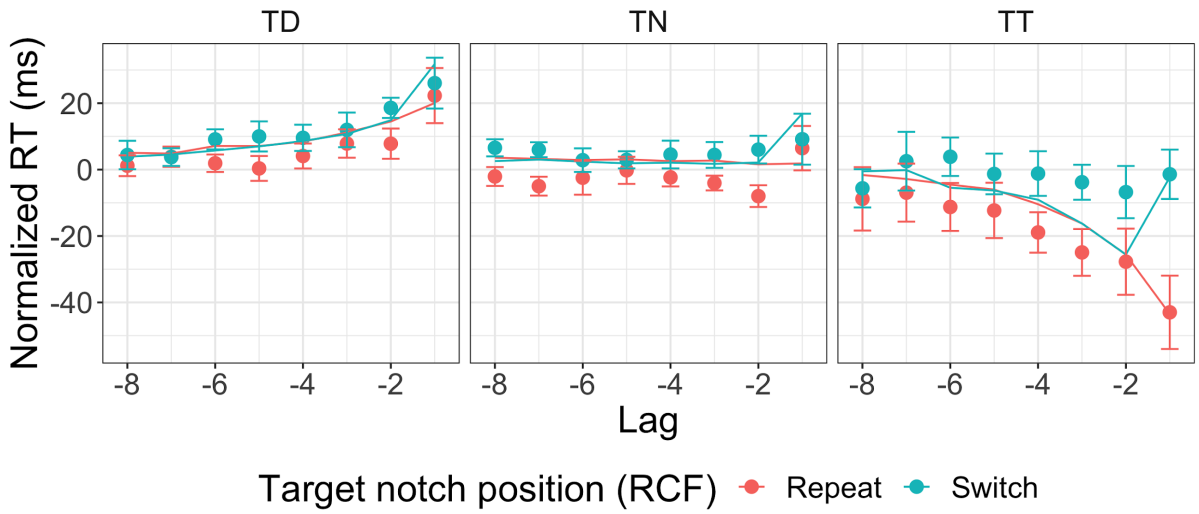

Fig AA**:** Temporal profile of the response-based inter-trial effects with model predictions based on the “PG step NDT” rule for response based updating**.**
