# Supplementary material for: Inter-trial effects in priming of pop-out: Comparison of computational updating models
Source: PLoS Comput Biol. 2021 Sep 3;17(9):e1009332. doi: 10.1371/journal.pcbi.1009332 (PMC8445473; doi:10.1371/journal.pcbi.1009332)
Supplement: S4 Appendix — (PDF) [file pcbi.1009332.s004.pdf]

S4 Appendix: Three stage model

The modeling framework we used for the model comparison was based on an evidence accumulation model with a single stage, that is, a single evidence accumulation process, though in reality each trial of the task likely involved more than one perceptual decision. It is therefore important to confirm that the best updating rules found in our model comparison still predicts the inter-trial effects well if they are applied to the parameters of a more realistic model involving multiple stages of perceptual decisions. In this appendix, we confirm this for a model with three different *stages*: a stage involving a decision about which item to attend to; a stage involving confirming whether the selected item is the target; and a stage involving making a response decision. The first two stages could be repeated multiple times if a wrong item is selected (i.e., selecting a distractor rather than the target). Each stage is modeled as an accumulation to threshold of a decision variable using diffusion process, that is, at each time step a random sample from a Gaussian distribution $N(\mu, \sigma)$ is accumulated to the decision variable, where μ determines the drift rate and σ the amount of random noise in the evidence accumulation process.

In the first stage, four accumulators, one for each item in the search display, competed for attentional selection, with the evidence accumulation continuing until one of the accumulators first reached a threshold $\boldsymbol{\theta}$, and the item selected being determined by which accumulator crossed the threshold first. The accumulator associated with the target position and the three associated with the three distractor positions had different drift rate parameters μ_T (for the target position) and μ_D (for each distractor position), but the same threshold $\boldsymbol{\theta}$ and noise parameter σ. The time required by the first stage was determined by the number of time steps before the first threshold crossing (for simplicity, we let each time step represent one millisecond).

The first (item-selection) stage was followed by the target-confirmation stage, representing the time needed to confirm whether the selected item was in fact the target. For simplicity, we assumed that the correct decision was always reached in the target-confirmation stage. The time used for the target-confirmation stage was modeled as the number of time steps before a single accumulator crossed threshold, with a different drift rate parameter μ_C, but the same threshold $\boldsymbol{\theta}$ and noise parameter σ as in the item-selection stage. If the target was selected in the item-selection stage, the target-confirmation stage was followed by the response-selection stage. If a distractor was selected, the selection and confirmation stages were first repeated until the target was selected, followed by the response-selection stage. Inhibition of return was modeled by excluding the accumulators associated with items that had previously been selected from the competition in repeated selection stages (consequently, the selection and confirmation stages were never repeated more than four times).

The response-selection stage was modeled as a single accumulation process which continued until either a positive or a negative threshold, $+\boldsymbol{\theta}$ or $-\boldsymbol{\theta}$, corresponding to a correct or incorrect

response decision, was reached, with drift rate μ_R and noise σ. Additionally, we considered the possibility that the response decision could be biased towards one response alternative by allowing the accumulation to start from a non-zero value represented by a starting point parameter S0.

The total response time (RT) on a trial was computed as the sum of the number of time steps required for each repetition of each stage (1-4 repetitions of the target-selection and confirmation stages and a single repetition of the response-selection stage) plus a non-decision time parameter $\boldsymbol{\tau}$. In total, the model has eight parameters: μ_T, μ_D, μ_C, μ_R, $\boldsymbol{\theta}$, σ, S0, and $\boldsymbol{\tau}$. The parameters were set to approximately match the mean RT across all participants and sessions, the standard deviation of RT averaged over all participants and sessions, and the average error rate. Additionally, we chose the parameter values to obtain approximately 40% of incorrect item decisions in the item-selection stage, based on the estimated frequency of such errors in Experiment 1 of Rangelov et al. [1]. Fig AB shows how the RT distribution of the model compares to those of the participants in the experiment (combining both sessions for each participant).

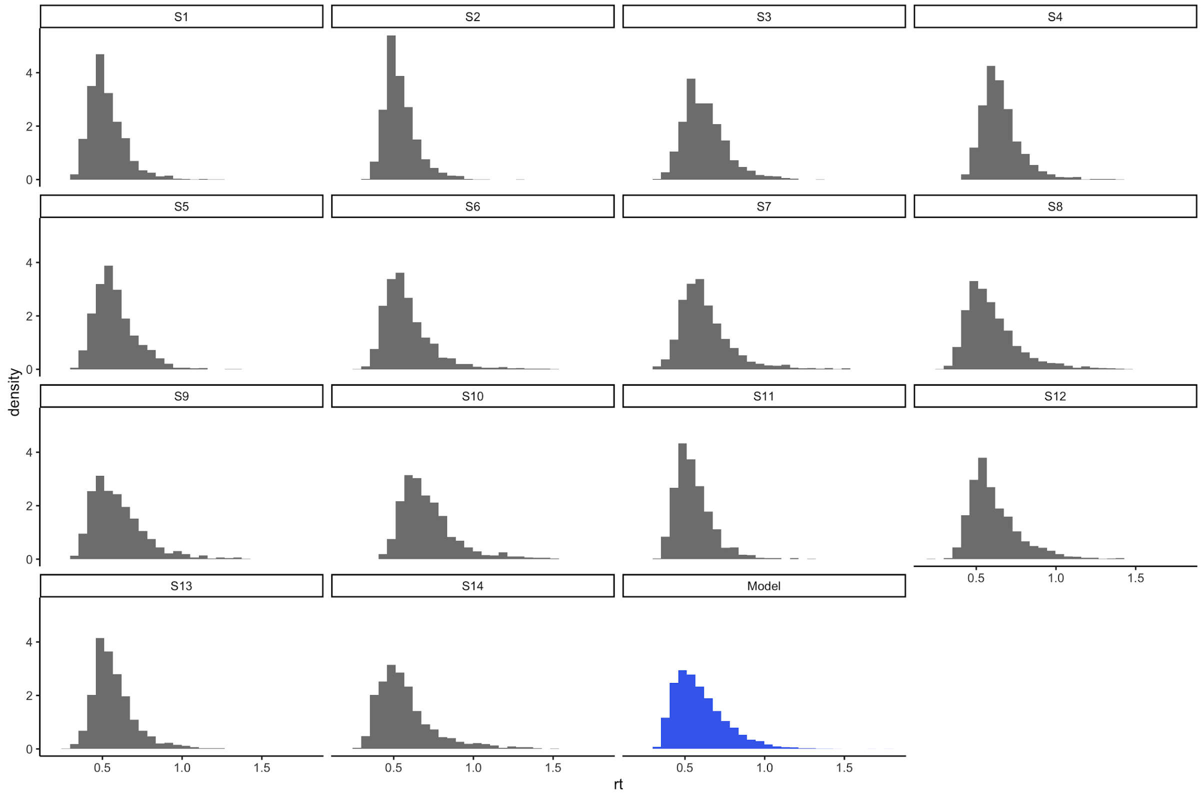

Fig AB: Response-time distributions for the three-stage model (bottom right, blue color) and the 14 participants in the experiment.

To test whether using the three-stage model, instead of the single-stage model, would significantly change the predicted pattern of inter-trial effects, we applied the winning updating rules from our model comparison to the parameters of the three-stage model. The winning rules for color-

and position-based inter-trial effects were both applied to the drift rate parameter associated with the target location (μ_T) in the first, item-selection, stage (in line with the evidence from the N2pc effects; see S5 Appendix). The winning rule for response-based inter-trial effects was applied to the starting point parameter (S0) in the third, response-selection, stage. We used the actual stimulus sequences, and the best updating rule parameters found with the single-stage model (with some modifications as described below), for each participant and session, to create the sequences of parameter values that were applied to the three-stage model. The memory parameter α of each updating rule as well as the parameters β_0 and $\boldsymbol{\omega}$ of the "Position-gradient Bayesian S0" updating rule was were used without modification. However, the parameters determining the size of weight updates, Δ for the color-based "Weighted rate" rule and Δ_t and Δ_d for target facilitation and distractor inhibition in the position-based "Weighted rate with distractor inhibition" rule were scaled by a factor of 4 to get approximately the right size of the inter-trial effects (details of the rules can be found in the main document). This scaling was necessary because in the three-stage rule, each stage represents only a smaller part of the total RT; so the same drift rate change applied to a parameter of one of the stages in the three-stage model would have less of an effect compared to the single-stage rule, where a single rate determines the full RT distribution. Since, for the three-stage model, we could only generate samples from the predicted RT distributions, compared to having an established mathematical function for the distribution (and therefore also the predicted mean RT) for the single-stage model, we computed the predictions for each participant and session ten times[1], in order to obtain a better estimate of the predicted mean. We averaged the predicted inter-trial effects over all stimulus sequences and repetitions.

Figs AC-AE below show how the predicted temporal profiles of inter-trial effects from the three stage model compared to the inter-trial effects found in the data. Because we did not fit the RT distributions of each individual participant with the three-stage model, rather used a single set of parameters to attempt to capture the "average" distribution, the predicted overall mean RT differed somewhat from the actual mean RT in the data. For these figures, we therefore computed the normalized model predictions slightly differently compared to those for the single-stage model (see Figs 9, 11 and 13 in the main document). Specifically, we focused on the inter-trial effects here by subtracting the overall mean predicted RT, rather than the overall mean RT from the data, to ignore individual differences in mean RTs (these differences between the single- vs. multi-stage models could in principle be gone by fine-tuning the parameters of the three-stage model to the RT distribution of each participant, which is not focus here).

[1] That is, the parameters of the three-stage model itself are based on averaged values, but then to obtain predictions for the inter-trial effects, trial-to-trial changes were applied to some of the parameters, based on the winning updating rules from the (single-stage) model comparison, and those trial-to-trial parameter changes were individualized for each participant, based on the updating rule parameters that we obtained with the single-stage model.

The predictions for color- and position-based inter-trial effects were very similar to those obtained with the single-stage model, and match the behavioral data well. Concerning response-based inter-trial effects, the predictions slightly underestimate the effects in the repeated target-position condition (but also overestimate the response-based effects slightly less in the other conditions, compared to the single-stage model), though they still closely match the pattern in the data and the predictions of the single-stage model.

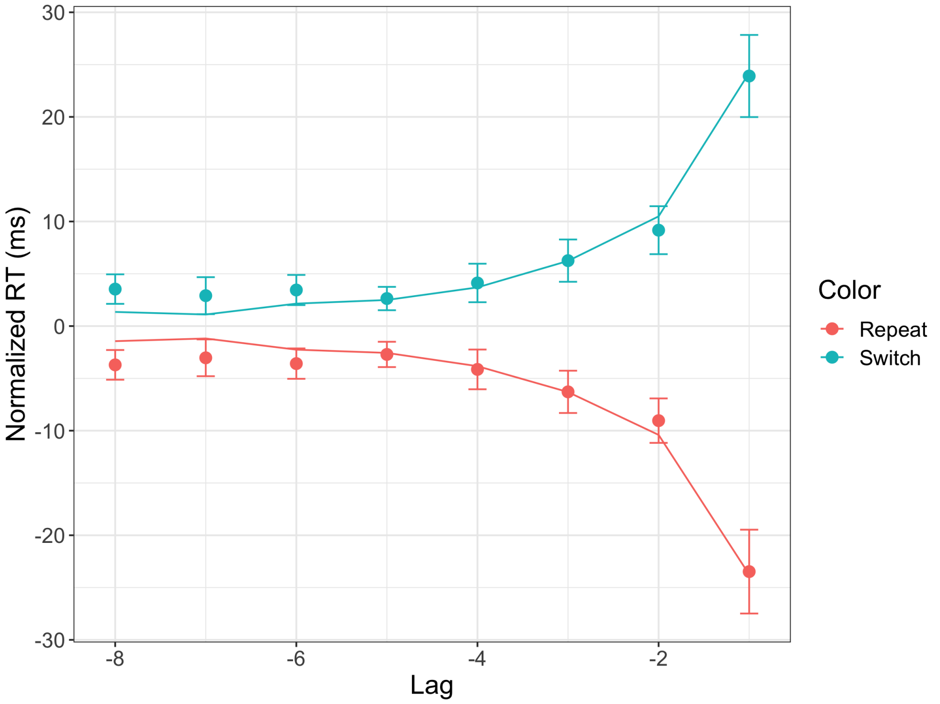

Fig AC: Temporal profile of the color-based inter-trial effects and the predictions of the three stage model. Filled circles depict the behavioral data, lines the model predictions. Error bars represent 95% confidence intervals.

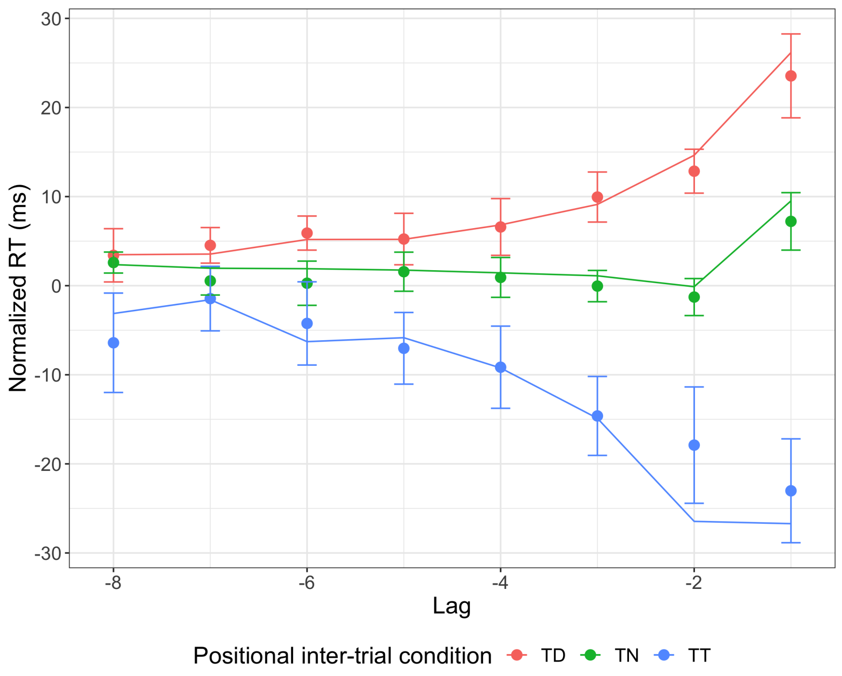

Fig AD: Temporal profile of the position-based inter-trial effects and the predictions of the three stage model. Filled circles depict the behavioral data, lines the model predictions. Error bars represent 95% confidence intervals.

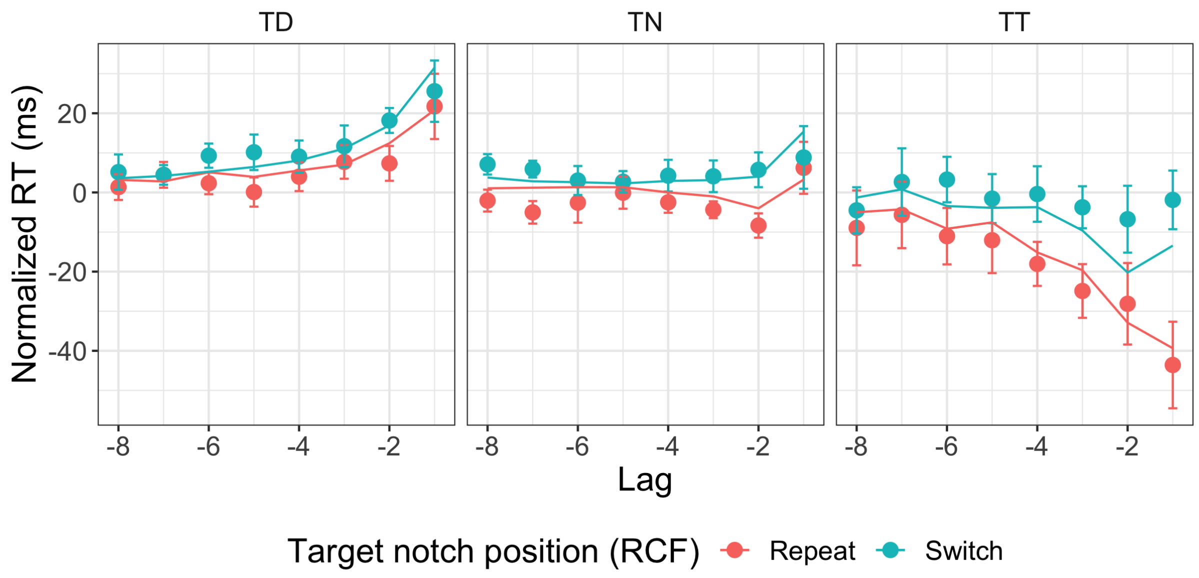

Fig AE: Temporal profile of the response-based inter-trial effects and the predictions of the three stage model. Filled circles depict the behavioral data, lines the model predictions. Error bars represent 95% confidence intervals.

Reference

1. Rangelov D, Müller HJ, Zehetleitner M. Failure to pop out: Feature singletons do not capture attention under low signal-to-noise ratio conditions. J Exp Psychol Gen. 2017;146: 651–671.
