# Supplementary material for: Inter-trial effects in priming of pop-out: Comparison of computational updating models
Source: PLoS Comput Biol. 2021 Sep 3;17(9):e1009332. doi: 10.1371/journal.pcbi.1009332 (PMC8445473; doi:10.1371/journal.pcbi.1009332)
Supplement: S5 Appendix — (PDF) [file pcbi.1009332.s005.pdf]

# S5 Appendix: Reanalysis of EEG data from Gökce et al. (2015)

The EEG data were obtained from Gökce et al., 2015 (*Frontiers in Psychology*) and analyzed anew for the present study in an attempt to evaluate main effects and interactions arising from color and positional repetitions on early visual processing.

Initial data were recorded from 64 Ag/AgCl electrodes at a sampling rate of 1000 Hz (10/10 system; American Electroencephalographic Society, 1994). The recorded EEG signals were amplified by BrainAmp DC amplifiers (Brain Products, Munich, Germany) using a 0.1- to 250-Hz band-pass filter. Electrophysiological signals were filtered offline with a 0.1–40-Hz band-pass (Butterworth 0 phase, 24 dB/Oct). All electrodes were referenced to FCz during recording and re-referenced offline to averaged mastoids. An infomax independent-component analysis was conducted to identify and back-transform blink and/or horizontal eye movement artifacts. Only trials with correct responses were included in the analysis. See Gökce et al. (2015) for more detail on EEG data collection and preprocessing.

For the PCN analyses, the EEG data were epoched into 500-msec periods preceded by a 200-msec pre-stimulus baseline and the data were baseline corrected. In order to isolate lateralized PCN difference waves from the non-lateralized ERPs, the waveforms at the electrodes PO7/8 ipsilateral to the side of the target location were subtracted from the contralateral ERPs. The PCN latencies were defined as the maximum negative deflection within the time windows 150–350 ms post-stimulus. PCN amplitudes were determined by averaging 5-sample points before and after the respective maximum deflections.

Grand average waveforms at PO7/PO8 electrodes and difference waves for same-color and different-color trials were separated for target-at-target (TT), target-at-neutral (TN), and target-at-distractor position conditions (TD; see Fig AF). Two-way repeated measures ANOVA comparing the amplitudes of the N2pc in the six (color: same, different; position: TT, TN, TD) cells of our experimental design revealed a significant main effect position, $F(2, 26) = 11.06$, $p < .001$, $\eta_p^2 = 0.46$.

Follow-up t-test comparing different levels of spatial positions to each other revealed that TN condition (-3.34 mV) produced *more* negative amplitude relative to TT condition (-2.85 mV), $F(1, 13) = 12.07$, $p = .004$, $\eta_p^2 = 0.48$), and *less* negative amplitude relative to TD condition (-3.86 mV), F(1,

13) = 4.66, p = .05, $\eta_p^2$ = 0.26. As can be seen in the Fig AF, the most pronounced difference was observed between the TT and TD conditions, F(1, 13) = 16.77, p = .001, $\eta_p^2$ = 0.56. On the other hand, the analysis of amplitudes resulted in non-significant main effect color, F(1, 13) = 1.16, p = .3, $\eta_p^2$ = 0.08, as well as insignificant interaction of color x position, F(2, 26) = 2.42, p = .108, $\eta_p^2$ = 0.16.

The latencies of the N2pc were also examined in a second color x position repeated-measured ANOVA which resulted in significant main effect of color, F(1, 13) = 33.19, p < .001, $\eta_p^2$ = 0.72. More precisely, preceding color-same trials (248.3 ms) resulted in speeded peak latencies relative to color-different trials (278.4 ms). Finally, the analysis of latencies also resulted in non-significant main effect of position, F(2, 26) = 1.39, p = .268, $\eta_p^2$ = 0.1, and non-significant interaction of color x position, F(2, 26) = 1.3, p = .29, $\eta_p^2$ = 0.09.

Taken together, these results demonstrate that both position and color of N-1 trials facilitate attentional selection in subsequent visual search.

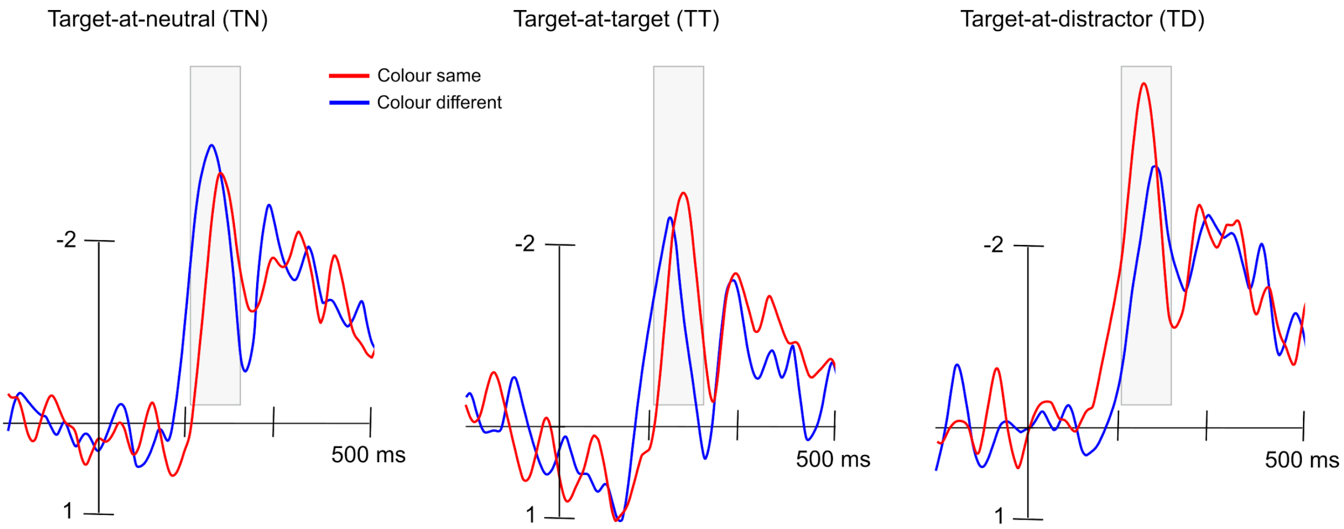


Fig AF: Lateralized ERP difference waves (PO7/8) plotted separately for position and colour conditions
