# Supplementary material for: Inter-trial effects in priming of pop-out: Comparison of computational updating models
Source: PLoS Comput Biol. 2021 Sep 3;17(9):e1009332. doi: 10.1371/journal.pcbi.1009332 (PMC8445473; doi:10.1371/journal.pcbi.1009332)
Supplement: S6 Appendix — (PDF) [file pcbi.1009332.s006.pdf]

S6 Appendix: Cross-validation - out-of-fold model predictions

In order to check to what extent the best-performing model in our model comparison was overfitting, we performed a cross-validation: for each block of trials, we evaluated the model based on the best parameters from fitting to the remaining blocks. In the cross-validation section of the main manuscript, we assess the degree of overfitting based on how the Akaike Information Criterion differs between the cross-validated out-of-fold predictions and the predictions of the model fitted to all the data (including the block it is evaluated on) for the best model and the no-updating model. Here, we illustrate the predicted temporal profiles of inter-trial effects based on the out-of-fold predictions. Fig AG shows the out-of-fold predictions for color priming (for comparison, see Fig 9 in the main manuscript, which depicts the model predictions for color priming without cross-validation), Fig AH shows the out-of-fold predictions for position priming (for comparison, see Fig 11 in the main manuscript), and Fig AI shows the out-of-fold predictions for response priming (for comparison, see Fig 13 in the main manuscript). Overall, the predictions differ little from those we obtained without cross-validation – suggesting that, while there may have been some degree of overfitting, our best updating rules capture regularities in the data in a way that generalizes across blocks.

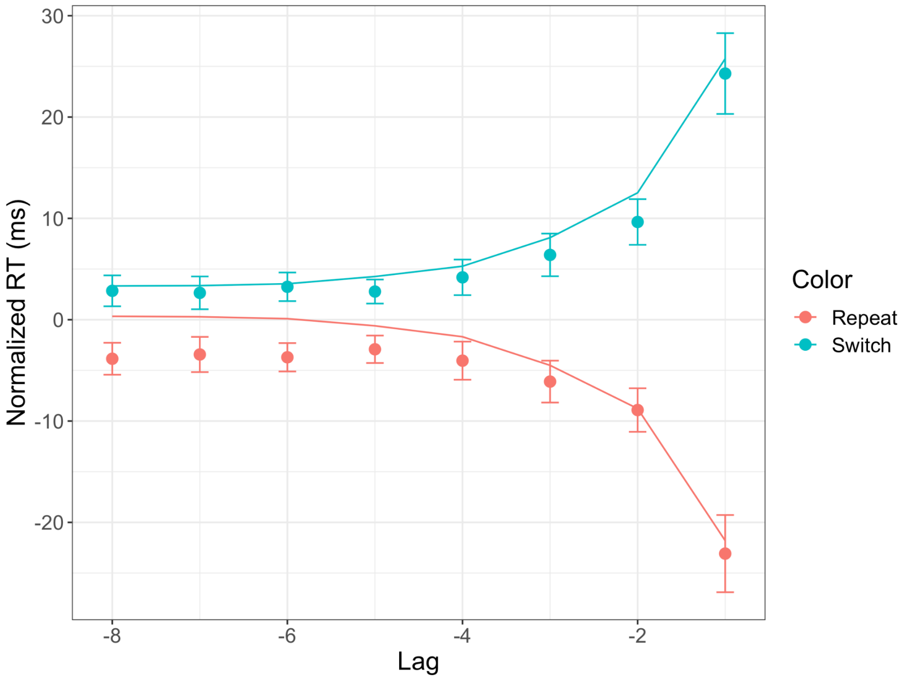

Fig AG: **Temporal profile of the color-based inter-trial effects and out-of-fold predictions.** Mean normalized RT for repeated vs. switched target color on (current) trial *n* compared to (preceding) trial *n-1* (lag 1), *n-2* (lag 2), etc. up to *n-8* (lag 8). Normalized RTs were first averaged across the two sessions for each participant; the resulting (individual mean normalized) RTs were then used to compute the overall means and confidence intervals, across participants. Filled circles depict the behavioral data, while lines show out-of-fold model predictions from the cross-validation. Error bars represent 95% confidence intervals.

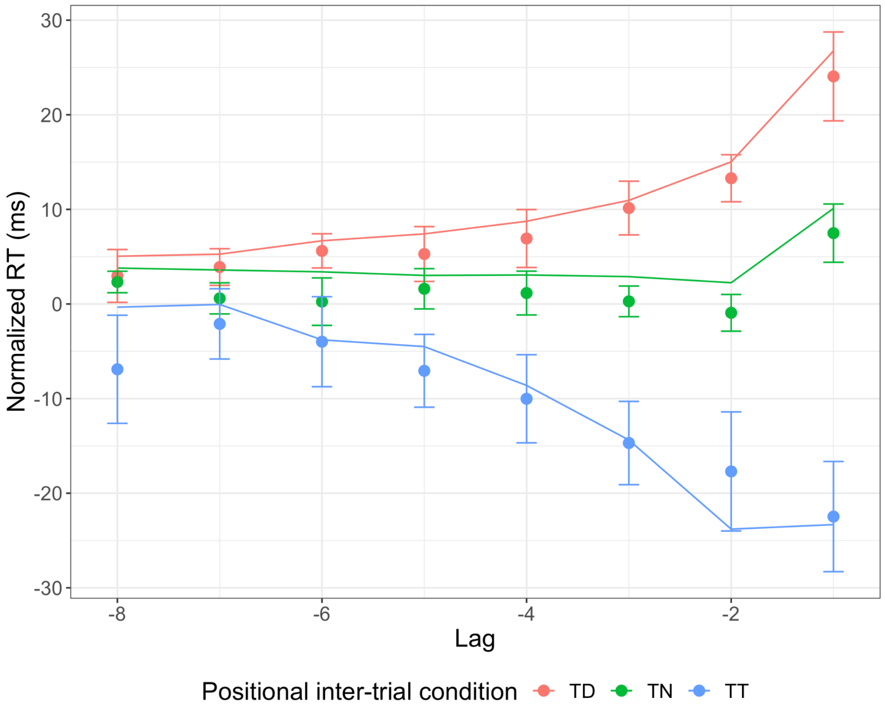

Fig AH: **Temporal profile of the position based inter-trial effects and out-of-fold predictions.** Mean normalized RT for different positional inter-trial conditions, target in previous target condition (TT), target in previous distractor position (TD), target in previously neutral (unoccupied) position (TN), compared to trial n-1 (lag 1), n-2 (lag 2), etc. up to n-8 (lag 8). Normalized RTs were first averaged across the two sessions for each participant; the resulting (individual mean normalized) RTs were then used to compute the overall means and confidence intervals, across participants. Filled circles depict the behavioral data, while lines show out-of-fold model predictions from the cross-validation. Error bars represent 95% confidence intervals.

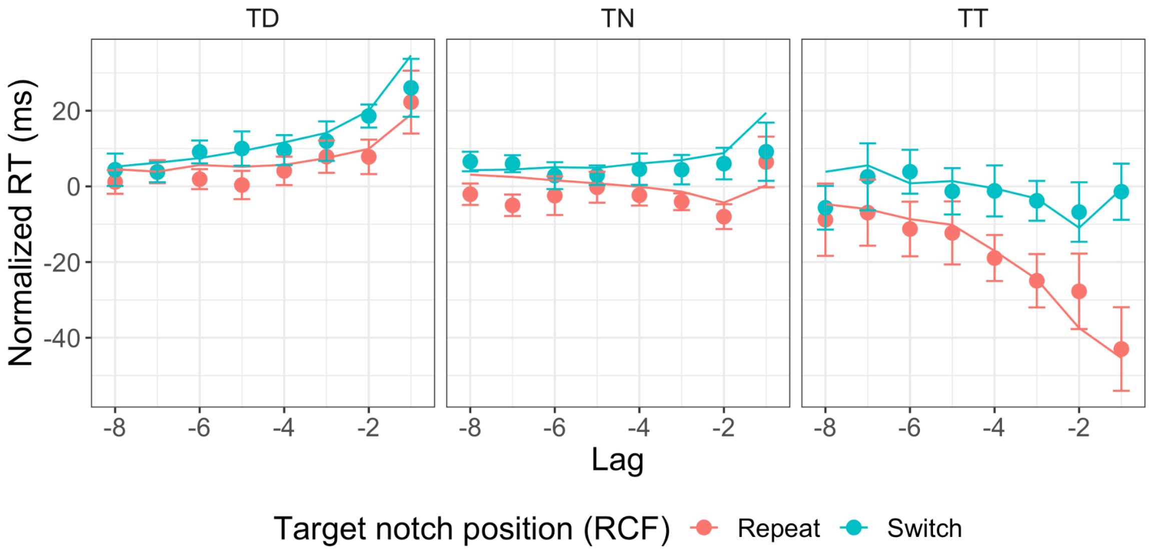

Fig AI: **Temporal profile of the response feature-based inter-trial effects and out-of-fold predictions.** Mean normalized RT for repetition vs. switch of the response defining target feature (notch on top or bottom of the diamond shape), compared to trial n-1 (lag 1), n-2 (lag 2), etc. up to n-8 (lag 8) for the different positional inter-trial conditions, target in previous target condition (TT), target in previous distractor position (TD), target in previously neutral (unoccupied) position (TN). Normalized RTs were first averaged across the two sessions for each participant; the resulting (individual mean normalized) RTs were then used to compute the overall means and confidence intervals, across participants. Filled circles depict the behavioral data, while lines show out-of-fold model predictions from the cross-validation. Error bars represent 95% confidence intervals. RCF: response-critical feature.
